# Validation of an interactive process mining methodology for clinical epidemiology through a cohort study on chronic kidney disease progression

**DOI:** 10.1038/s41598-024-79704-5

**Published:** 2024-11-14

**Authors:** Kaile Chen, Farhad Abtahi, Juan-Jesus Carrero, Carlos Fernandez-Llatas, Hong Xu, Fernando Seoane

**Affiliations:** 1 Department of Clinical Science, Intervention and Technology, Karolinska Institutet, Stockholm, 17177 Sweden; 2 Department of Biomedical Engineering and Health Systems, School of Engineering Sciences in Chemistry, Biotechnology and Health, KTH Royal Institute of Technology, Huddinge, 14157 Sweden; 3 Department of Clinical Physiology, Karolinska University Hospital, Stockholm, 17176 Sweden; 4 Department of Medical Epidemiology and Biostatistics, Karolinska Institutet, Stockholm, 17177 Sweden; 5 Division of Nephrology, Department of Clinical Sciences, Danderyd Hospital, Karolinska Institutet, Stockholm, 17177 Sweden; 6 SABIEN, ITACA, Universitat Politécnica de Valencia, Valencia, Spain; 7 Division of Clinical Geriatrics, Department of Neurobiology, Care Sciences and Society (NVS), Karolinska Institutet, Stockholm, 17177 Sweden; 8 Department of Medical Technology, Karolinska University Hospital, Stockholm, 17176 Sweden; 9 Department of Textile Technology, University of Borås, Borås, 50190 Sweden

**Keywords:** Process mining, Observational epidemiology study, Multistate model, Chronic kidney disease progression, Methodology, Chronic kidney disease, Risk factors, Statistical methods, Epidemiology, Data mining

## Abstract

Process mining holds promise for analysing longitudinal data in clinical epidemiology, yet its application remains limited. The objective of this study was to propose and evaluate a methodology for applying process mining techniques in observational clinical epidemiology. We propose a methodology that integrates a cohort study design with data-driven process mining, with an eight-step approach, including data collection, data extraction and curation, event-log generation, process discovery, process abstraction, hypothesis generation, statistical testing, and prediction. These steps facilitate the discovery of disease progression patterns. We implemented our proposed methodology in a cohort study comparing new users of proton pump inhibitors (PPI) and H2 blockers (H2B). PPI usage was associated with a higher risk of disease progression compared to H2B usage, including a greater than 30% decline in estimated Glomerular Filtration Rate (eGFR) (Hazard Ratio [HR] 1.6, 95% Confidence Interval [CI] 1.4–1.8), as well as increased all-cause mortality (HR 3.0, 95% CI 2.1–4.4). Furthermore, we investigated the associations between each transition and covariates such as age, gender, and comorbidities, offering deeper insights into disease progression dynamics. Additionally, a risk prediction tool was developed to estimate the transition probability for an individual at a future time. The proposed methodology bridges the gap between process mining and epidemiological studies, providing a useful approach to investigating disease progression and risk factors. The synergy between these fields enhances the depth of study findings and fosters the discovery of new insights and ideas.

**Supplementary Information:**

The online version contains supplementary material available at 10.1038/s41598-024-79704-5.

## Introduction

The inception of process mining within healthcare traces back to its initial utilisation in 2001 ^1^. As the amount of clinical data grew, it became popular to identify bottlenecks and regulatory adherence within healthcare processes^2^. The momentum behind its evolution has been particularly pronounced in clinical pathway investigations^3,4^, wherein it illuminates the operational intricacies of healthcare procedures, thereby furnishing invaluable real-world insights. This paradigm mirrors the extraction of a schematic process from extensive real-world data, giving an expeditious panoramic representation of data patterns. Furthermore, process mining provides various tools encompassing visualisation and data descriptive analysis^5^. Nevertheless, its advantages remain unrealised in the domain of longitudinal epidemiology studies despite the apparent applicability of process mining principles in this context.

Observational longitudinal epidemiology studies cover the prolonged observation of individuals or populations, aiming to comprehend the disease evolution and progression^6^. Classic observational epidemiology studies often focus on the relationship between exposure and a single event. For instance, survival analysis is a statistical method commonly used in epidemiology to analyse time-to-event data^7^. It is useful when studying transitions between exposure and outcome, such as the progression from baseline to a subsequent state. Such scenarios conventionally lend themselves well to established analytical models like the Cox regression or logistic models. However, the research landscape expands when the focus encompasses not only one outcome but also the intermediate events. For example, consider scenarios where a secondary progression or mortality occurs after the initial progression. When intermediate events also hold significance for an epidemiological study, process mining emerges as a useful investigative tool capable of probing and understanding complicated multistate events.

While process mining has shown promise in healthcare, its application to longitudinal epidemiology is limited. This study aims to fill this gap by proposing a methodology for employing process mining in observational epidemiology studies, specifically for investigating the impact of medications and comorbidities on kidney function. The proposed methodology offers a multistate analysis of disease progression, which extends beyond traditional approaches that focus on single outcomes. This methodology allows for the exploration of intermediate states and transitions, thereby providing a more comprehensive understanding of disease dynamics.

The novelty of this study lies in the integration of process mining into longitudinal epidemiology, offering a new perspective on analysing multistate disease progression. We applied this methodology to investigate the association between Proton Pump Inhibitors (PPI) and kidney function trajectories, given emerging evidence suggesting their potential nephrotoxicity and acceleration of kidney function decline^8–10^. The current study is particularly relevant given the widespread prescription of PPI and the importance of understanding its long-term impact on kidney function. Furthermore, the present study assessed the association of comorbidities, including gastrointestinal diseases, cardiovascular/cerebrovascular diseases, diabetes mellitus, and chronic obstructive pulmonary disease (COPD), with kidney function progression. To achieve this goal, we conducted a retrospective cohort study using the same data source as referenced in the study^11^ to validate whether the results obtained through our proposed methodology are comparable to those derived from conventional epidemiological methods.

The structure of the paper is as follows, Sect. 2 outlines the proposed methodology in detail. Section 3 presents the findings of a cohort study conducted using this methodology, and Sect. 4 discusses the practical implications, strengths, and limitations of applying this methodology to real-world data. Finally, Sect. 5 provides the study’s conclusion.

## Methodology

**Fig. 1:**
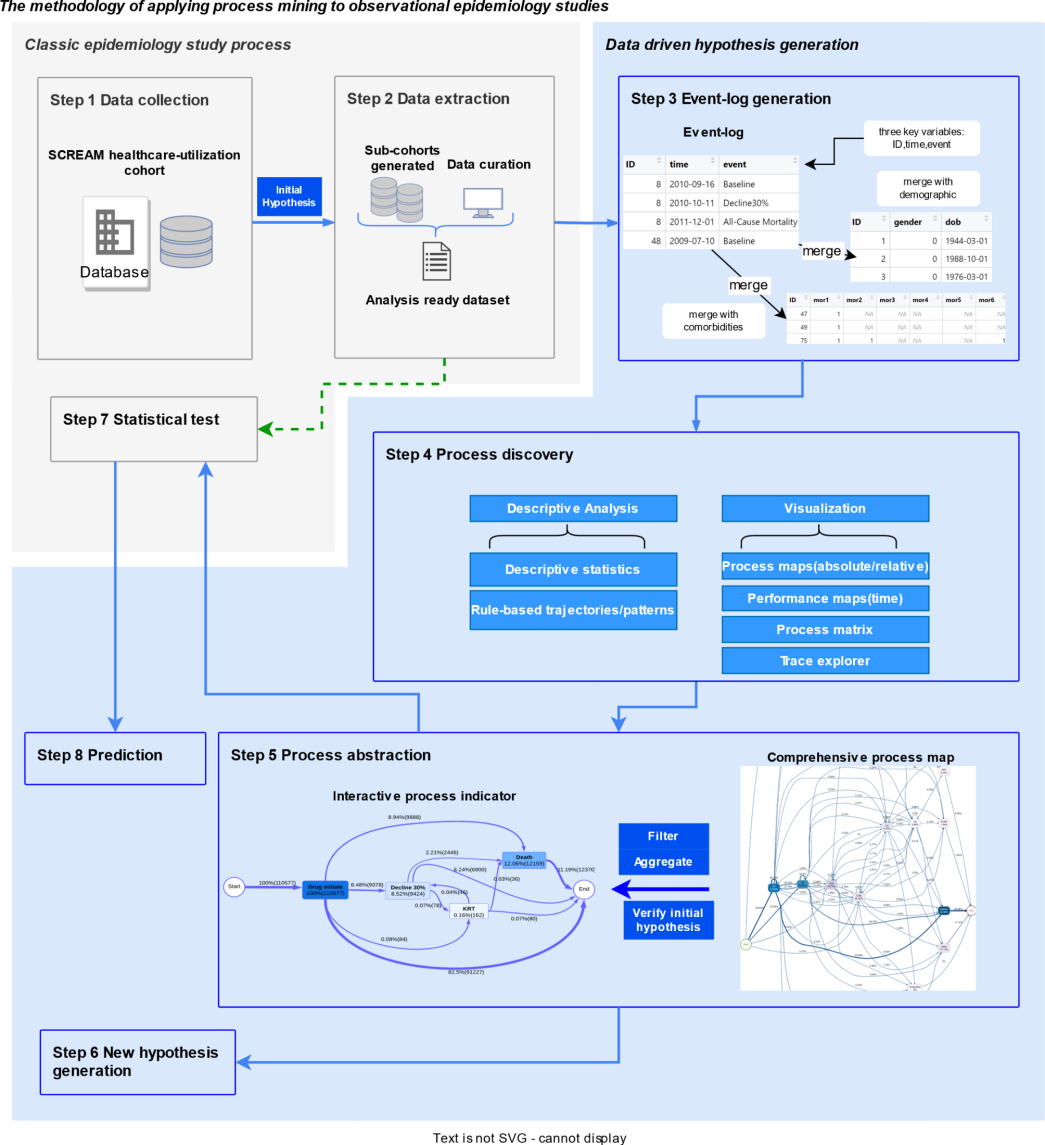
Proposed methodology workflow of process mining applied to observational epidemiology studies: The solid arrow illustrates the case study explored in this paper. The green dotted arrow represents the classic epidemiological study process, which is not performed in the current study.

We proposed a framework for applying process mining discovery in observational longitudinal epidemiology studies involving eight steps, as shown in Fig. 1. The workflow consists of two main parts: the traditional epidemiological study process is shown in grey, and the blue section signifies data-driven process mining. All the steps are described specifically in Sect. 2.1 to 2.8.

### Real-world database and setting

The first step in this methodology is to have a relevant real-world database that contains information about the event of interest, including timestamps for each event occurrence.

In this study, we used data from the Stockholm CREAtinine Measurements (SCREAM) database^12^ includes comprehensive laboratory and healthcare data from over 1.1 million adult residents in Stockholm, collected from 2006 to 2011, focusing on chronic kidney disease and associated healthcare outcomes. Medications are identified by recorded pharmacy dispensations through the Anatomical Therapeutic Classification (ATC) codes from linkage with the Swedish Prescribed Drug Registry. This study was conducted in accordance with the Declaration of Helsinki. Approval for the study was obtained from the Swedish National Board of Welfare and the Stockholm Regional Ethics Review Board, who deemed that informed consent was not required and provided de-identified data.

### Data extraction and curation

Data preprocessing, including cleaning and transformation, is a critical initial step to render the dataset compatible with process mining techniques. After preprocessing, the “analysis-ready dataset” necessitates including at least three key variables: patient ID, event or state, and the corresponding event occurrence time.

In this study, we built a retrospective cohort of new users of PPI and H2 blockers (H2B) from SCREAM. The index date was defined as the first dispensation date of PPI or H2B between 2007 and 2010. We included individuals who had newly initiated PPI or H2B and had at least a recorded serum/plasma creatinine test in connection with an outpatient consultation no more than one year before the index date. The definitions follow the same approach as previously published from the same data source^11^, which uses traditional statistical methods to compare the reproducibility of findings with the proposed process mining approach. New users were thus defined as the first-time PPI or H2B dispensed between January 1, 2007 (records in 2006 were used to ensure at least 12 months with no recorded use of these medications) and December 31, 2010 (records in 2011 were used to ensure at least one year follow up time to detect the effect); Individuals were excluded if (a) estimated glomerular filtration rate (eGFR) < 15 mL/min/1.73 m^2^ (The calculation of eGFR used the Chronic Kidney Disease Epidemiology Collaboration (CKD-EPI) 2009 creatinine Eq.(^13^); and (b) age < 18 years old.

The transition states were defined as (1) Drug (PPI/H2B) initiation; (2) an eGFR decline > = 30% relative to baseline eGFR, which defines a clinically meaningful drop in eGFR that has been accepted as a surrogate endpoint of CKD progression by regulatory agencies of drug approvals^14^; (3) Kidney replacement therapy (KRT), which is ascertained by linkage with the Swedish Renal Registry and includes both the date of kidney transplant and the date of start of maintenance dialysis; (4) All-cause mortality. If no events were observed by the end of the study (2011-12-31) or the participant emigrated from the Stockholm region, they were labelled as censored.

Independent covariates were ascertained on the index date, including age, gender and baseline eGFR. Baseline eGFR was defined as the most recent measurement before or on the PPI/H2B dispensation date. Time-dependent covariates included comorbidities and concomitant medications. Time-dependent comorbidities were identified based on diagnostic records available up to and including the event date. This study considered the following comorbidities as potential indications for acid-suppressive therapy: gastroesophageal reflux disease, Barrett’s esophagus, ulcer disease, helicobacter pylori infection, and upper gastrointestinal bleeding. Additionally, we investigated the following comorbidities to assess their influence: hypertension, diabetes mellitus, myocardial infarction, congestive heart failure, peripheral vascular disease, cerebrovascular disease, and COPD. Time-dependent concomitant medications were extracted based on dispensation records from the six months preceding each event. These medications included nonsteroidal anti-inflammatory drugs (NSAIDs)/aspirin, statins, and antithrombotics. The ICD-10 codes of included comorbidities and the ATC codes of PPI/H2B were summarised in Supplementary Table 1. Data management was conducted using R x64 4.1.2.

### Event-log generation

Process mining event log was generated based on the analysis-ready dataset, in which each row represents a single event and a single timestamp.

In our case study, we uploaded the analysis-ready dataset to R and employed the event-log generation function provided by an R package *bupaR*^15^ to produce the event-log necessary for subsequent process model development. For further analysis, we can merge the demographic or comorbidities dataset to the event-log when necessary. Other packages or applications, such as PmineR^16^, PMApp^17^, and ProM^18^, could also be employed for this purpose.

### Process discovery

Process discovery^5^ is the critical technique used to depict the process from the data into graphical workflows. A process discovery algorithm or miner serves to derive the process model. In the healthcare domain, prevalent process discovery algorithms/miners encompass “PALIA”, “Inductive Miner”, and “Care Flow Miner”^5^. Utilising the process model, we can conduct descriptive analyses, such as descriptive statistics and rule-based patterns, and visualise the process maps, process matrix, and trace explorer.

In the current case study, the process discovery algorithm/miner employed was the Directly-Follows Graph (DFG), which is a key component of the *bupaR* package^15^. This algorithm/miner facilitates the extraction of process models based on the time-sequential relationships between activities within an event log.

### Process abstraction

The complexity of disease event logs often results in initial process models exhibiting complicated relationships between events and traces, occasionally resembling a process map of high complexity commonly known as a ‘spaghetti’ pattern^19^. Such complexity presents significant challenges for researchers attempting to understand the process and derive meaningful insights from its elaborate configuration. The process abstraction aims to turn the process map mined in the process discovery process into a simpler, understandable and focused process model that can be used to study the process under analysis.

Consequently, using interactive process indicators (IPIs)^20^ for abstracting the process can provide a clear, human-understandable view by focusing on the essential parts of the process. A process indicator (PI) is defined as a visual representation of a process, i.e.,* a process map*, functioning as an indicator to measure and comprehend that process^20^. IPIs emerge from applying the interactive paradigm in collaboration with domain experts to generate process indicators^20^. Some strategies, such as sampling^21^, clustering^22^, and a deep learning-based method^23^, can be used to abstract simpler process models from complex event data with acceptable precision. One of the most commonly used techniques for abstraction is filtering^24^, which allows the selection of relevant events and pathways, thereby focusing on the key aspects of the process. Filtering can also be employed to stratify PIs for different patient subgroups based on their characteristics, enabling comparative analysis. Moreover, filtering helps reduce noise by eliminating irrelevant data. Another strategy is aggregation, where multiple distinct events are combined into a higher-level event, simplifying the process model without sacrificing critical information.

In the context of our case study, we applied filtering to the primary process model to stratify PIs for patients based on their use of PPI or H2B. The specific events representing key stages of disease progression were confirmed by an expert in clinical kidney disease epidemiology. We further aggregated two events (CKD Decline by 30%, KRT) into a single event (Decline 30% or KRT) for statistical modelling due to the limited number of KRT events. The choice of abstracting IPIs through filtering and aggregation was guided by the research question and clinical relevance. This approach ensured that no data were wasted while maintaining an accurate reflection of the real-world disease progression pathways. Additionally, filters were applied to stratify IPIs based on comorbidities, allowing us to further evaluate the association between comorbid conditions and kidney function progression trajectories.

### New hypothesis generation

Beyond the initial hypotheses, the process model analysis derived from the previous step 2.5, may provide new insights from the data.

### Test the hypothesis with the statistical method

The process model offers valuable visualisations and parameters to primarily verify the initial hypotheses. Statistical tests or models based on the data characteristics were conducted to ensure a better understanding and meaningful interpretation of the results.

In this case study, baseline characteristics were presented as mean and standard deviations for continuous variables or median and interquartile range (IQR) for continuous variables but with skewed data.

A multistate model was constructed based on the process model obtained in 2.4 to quantify and interpret the observed transitions comprehensively. This model was formulated as a continuous-time Markov process for modelling CKD progression, under the Markov assumption that future transitions depend only on the current state. The Markov assumption is considered suitable for modelling CKD progression because, in clinical practice^25^, nephrologists and clinical guidelines emphasise the current eGFR stage when determining treatment strategies. Additionally, previous studies^26,27^ on CKD progression have used multistate Markov models to estimate progression rates and identify risk factors, providing support for the appropriateness of this assumption in the current context. We later incorporated time-dependent covariables to relax the assumption of time-homogeneous, allowing for transition rates that may vary over time, better capturing the complexity of CKD progression. The multistate model developed in this study incorporated covariates, as outlined in Sect. 2.2, to account for their potential influence on disease progression transitions. Adjusted hazard ratios, along with corresponding 95% confidence intervals (CIs), were used to estimate the effects of these covariates on the transition probabilities between disease states. The multistate model in this study is constructed as follows:

$$\displaylines{\:{q_{rs}}\left( X \right) = {q_{rs}}\left( 0 \right)*\exp (\beta \:1*{x_{treatment\left( {1 = ppi,\:0 = h2b} \right)}} + \beta \:2*{x_{age\:group}} + \cr \beta \:3*{x_{gender\left( {1 = female,\:0 = male} \right)}} + \:\beta \:4*{x_{gastrointestinal\:disease\left( {1 = yes,\:0 = no} \right)}} + \cr \beta \:5*{x_{cardiovascular\:and\:cerebrovascular\left( {1 = yes,\:0 = no} \right)}} + \beta \:6*{x_{diabetes\left( {1 = yes,\:0 = no} \right)}} + \cr \beta \:7*{x_{COPD\left( {1 = yes,\:0 = no} \right)}} + \beta \:8*{x_{NSAIDS\left( {1 = yes,\:0 = no} \right)}} + \:\beta \:9*{x_{Statins\left( {1 = yes,\:0 = no} \right)}}) + \cr \beta \:10*{x_{Antithrombotics\left( {1 = yes,\:0 = no} \right)}} + \:\beta \:11*{x_{eGFR\:baseline\left( {0 = G1,\:1 = G2,\:3 = G3A,\:4 = G3B,\:5 = G4} \right)}} \cr}$$

The *msm*^28^ package for multistate modelling in R was used. Data analysis was conducted using R x64 4.1.2.

### Prediction

The transition intensity of the process model was quantified using a multistate model in step 2.7. After deriving this multistate model, we were able to predict a specific event/state at a future time or a given individual^28^.

In our case study, we developed a “Risk prediction” tool (https://transitionprobability.shinyapps.io/Risk_Prediction/) based on the process model and multistate model using R shiny 1.7.5.

## Results

**Fig. 2:**
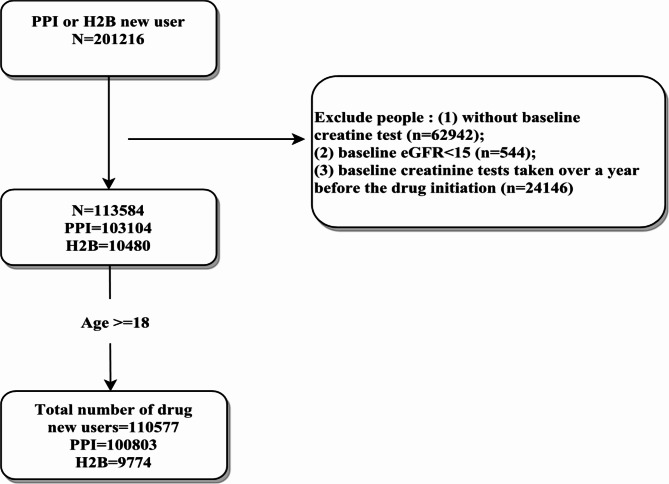
Flowchart of data collection for the cohort comprising new users of Proton Pump Inhibitors (PPI) and H2 blockers (H2B).

**Fig. 3:**
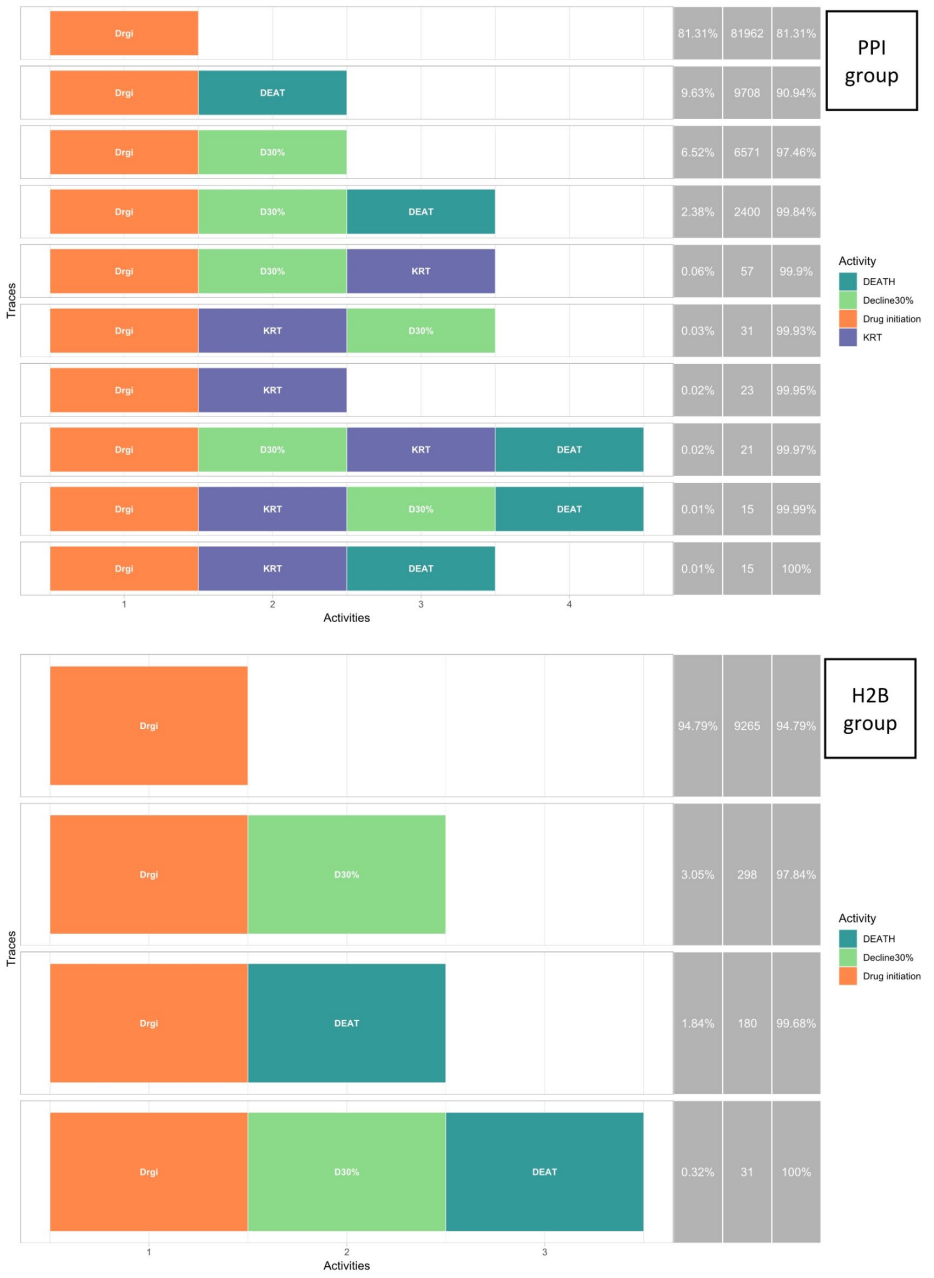
Distribution of distinct traces between the PPI and H2B groups. The statistics on the right side of the figure represent the relative frequency, absolute frequency, and cumulative relative frequency for each trace.

Overall, 110,577 subjects were included in the analysis; 100,803 initiated PPI therapy, and 9,774 initiated H2B (Fig. 2). An event log was created based on these subjects, with a total of 132,533 events recorded—122,219 events in the PPI group and 10,314 events in the H2B group. The PPI group exhibited 10 distinct traces, whereas the H2B group had 4 distinct traces. A summary of the distinct traces between the PPI and H2B groups is provided in Fig. 3.

As shown in Table 1, PPI users were older and consisted of more males than H2B users. Regarding comorbidities, PPI users exhibited a higher prevalence. Concerning baseline eGFR, PPI users displayed a greater proportion of individuals classified as having lower eGFR values than H2B users.

**Table 1:** Population characteristics of baseline between new users of PPI and H2B.

	PPI, *n* = 100,803	H2B, *n* = 9774
**Age**,** median (IQR)**	59 (44,72)	48 (34,63)
**Age group**,** n%**		
18 ~ 45	27,401 (27.2)	4273 (43.7)
46 ~ 64	34,487 (34.2)	3083 (31.5)
65 ~ 80	26,327 (26.1)	1732 (17.7)
>=81	12,096 (12)	532 (5.4)
**Gender**,** n%**		
Female	60,046 (60)	6193 (63)
Male	40,757 (40)	3581 (37)
**Comorbidity**,** n%**		
**Gastrointestinal diseases**	11,634 (11.5)	680 (7)
Gastroesophageal reflux disease	6552 (6.5)	486 (5)
Upper gastrointestinal tract bleeding	1701 (1.7)	79 (0.8)
Ulcer disease	4273 (4.2)	141 (1.4)
*H. Pylori* infection	1701 (1.7)	79 (0.8)
**Cardiovascular and** **cerebrovascular diseases**	49,736 (49.3)	3338 (34.2)
Myocardial infarction	5669 (5.6)	318 (3.3)
Cerebrovascular disease	7749 (7.7)	424 (4.3)
Peripheral vascular disease	3491 (3.5)	168 (1.7)
Congestive heart failure	3881 (3.9)	184 (1.9)
Hypertension	48,035 (47.7)	3215 (32.9)
**Diabetes mellitus**	12,634 (12.5)	902 (9.2)
**Chronic obstructive pulmonary disease (COPD**)	13,298 (13.2)	1064 (10.9)
**Concomitant medication**,** n%**		
NSAIDs, aspirin	30,716 (30.5)	1992 (20.4)
Statins	18,444 (18.3)	1235 (12.6)
Antithrombotics	27,745 (27.5)	1404 (14.4)
**eGFR(mL/min/1.73m**^**2**^**)**,** median (IQR)**	47.8(38.2,54.6)	50.6(42.4,56.2)
**eGFR categories**,** n%**		
G1 ( > = 90 mL/min/1.73m^2^)	51,759 (51.3)	6320 (64.7)
G2 (60–89 mL/min/1.73m^2^)	37,675 (37.4)	2882 (29.5)
G3A (45–59 mL/min/1.73m^2^)	6837 (6.8)	409 (4.2)
G3B (30–44 mL/min/1.73m^2^)	3385 (3.4)	135 (1.4)
G4 (15–29 mL/min/1.73m^2^)	1147 (1.1)	28 (0.3)

### Process mining to describe the possible impact of medication and comorbidities in real-world kidney function progression

**Fig. 4:**
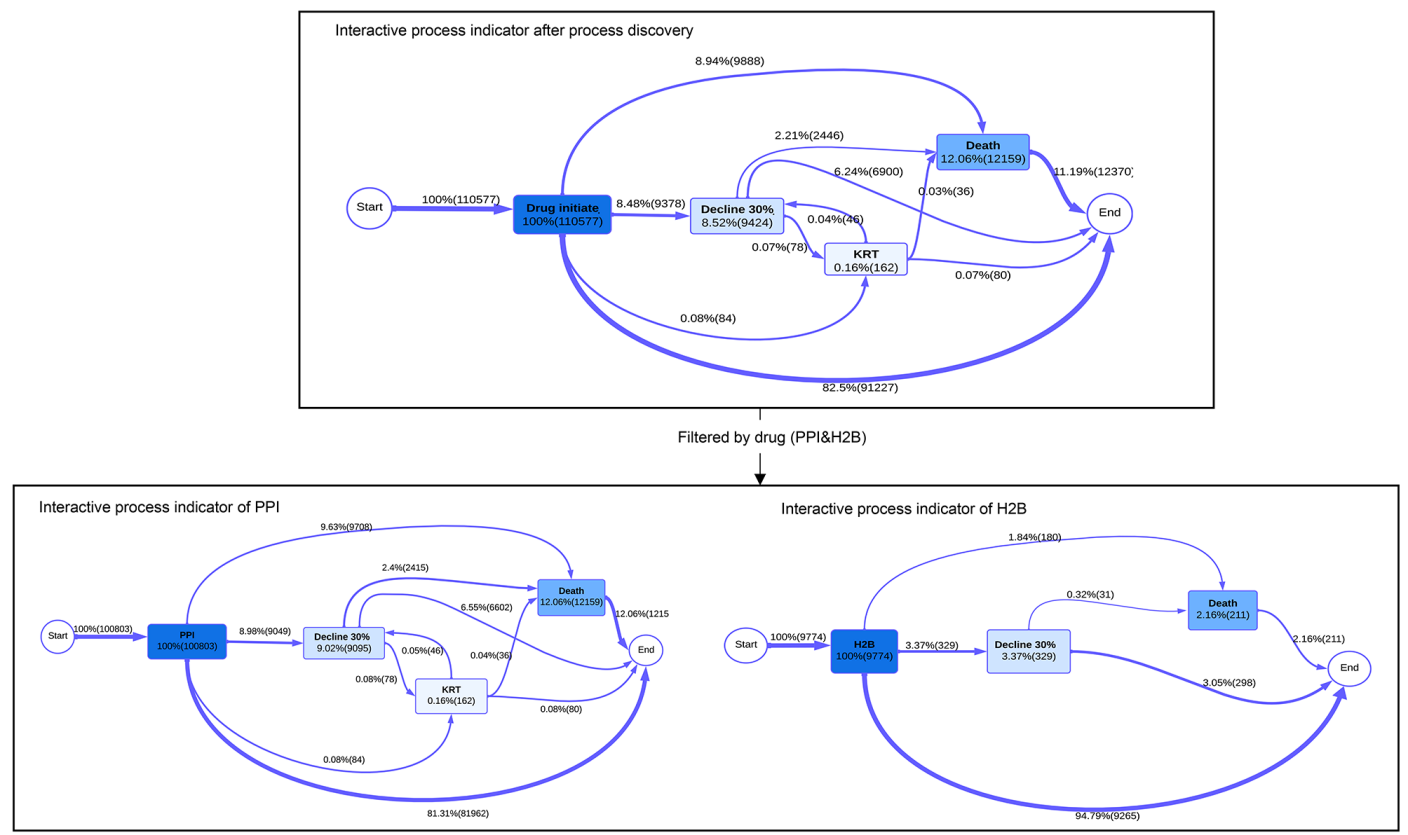
Interactive process indicators; the nodes are the events/states with darker color means the more events frequency; Arrows represent time-ordered sequences of traces; the thickness of these arrows corresponds to the frequency of occurrence.; the definition of each node are: “Drug Initiate” - start date for Proton Pump Inhibitors (PPI) or Histamine-2 Blockers (H2B); “Decline30%” − 30% or more reduction in baseline kidney function (eGFR); “KRT” - Kidney Replacement Therapy (includes transplant and dialysis, as per Swedish Renal Registry); “Death” - all-cause mortality. Figure created using flowchart tool to simulate results consistent with those obtained from “bupaR”. Supplementary Fig. 1 depicts the original process flow as generated by the ‘bupaR’ package.

The process indicators for PPI and H2B were generated (Fig. 4 & Supplementary Fig. 1), revealing notable disparities in the proportions of events and transitions between the two groups. Upon comparing process indicators between PPI and H2B users, it was observed that PPI users exhibited a higher prevalence of a 30% decline in eGFR, with 9.02% compared to 3.37% in H2B users. Additionally, all-cause mortality was higher among PPI users (12.06%) than in the H2B group (2.16%). Notably, the incidence of KRT was 0.16% in the PPI group, while no cases of KRT were recorded in the H2B group. These discrepancies suggested a possible negative impact of PPI on kidney function.

Process discovery can be used to generate the process indicator with the filtering technique. Several filters based on the baseline of age, gender, and comorbidities were configured. The process indicator allows for the assessment of covariates in relation to different events and transitions.

Furthermore, individuals with comorbidities such as gastrointestinal diseases, cardiovascular/cerebrovascular diseases, diabetes, and COPD are more prone to experience a *30% eGFR decline*, *KRT* and *All-cause mortality* compared to those without these conditions (shown in Supplementary Fig. 2 ~ 8).

### Overall comparison among different transitions

We observed a limited number of *KRT* events (Fig. 4), so a combination of the *Decline 30%* with *KRT* were undertaken to enhance the fit for the multistate model since the constrained number of events has led to the inadequacy of the multistate model. The multistate model was then built on three events which were *Drug initiation*, *Decline 30%/KRT*, and *All-cause mortality*.

We incorporated all relevant independent and time-dependent covariates that were previously supported and validated through the process discovery step. Table 2 presents the adjusted hazard ratios (HRs) for all covariates and treatments in each of the three transitions from the multistate model. The findings indicated that multistate models pinpoint the significance of covariates for specific transitions.

In the transition from ‘Drug initiation’ to ‘Decline 30%/KRT’, all provided covariates exhibited statistically significant associations with this transition. Increased hazards of experiencing a *30% decline* in *eGFR* were observed in individuals using PPI, those of older age, individuals with gastrointestinal diseases, cardiovascular/cerebrovascular diseases, diabetes mellitus, COPD, utilisation of antithrombotics and those with higher eGFR categories. Conversely, female gender and the utilisation of NSAIDs or statins were associated with reduced hazards of this transition. (Table 2)

Regarding the transition from ‘Drug initiation’ to ‘All-cause mortality’, increased hazards were associated with PPI usage, older age, diabetes, COPD, and utilisation of antithrombotics. Conversely, female gender, with gastrointestinal diseases, and the utilisation of NSAIDs or statins were statistically associated with a decreased risk of this transition (Table 2).

In the transition from ‘Decline 30%/KRT’ to ‘All-cause mortality’, increased risks were associated with PPI usage, older age, cardiovascular/cerebrovascular diseases, and COPD, while being female and the utilisation of NSAIDs or statins were associated with a decreased risk of this transition (Table 2).

**Table 2:** Comparison of progression transitions among covariates using adjusted Hazard ratios (HR) and corresponding 95% confidence interval (CI).

	Drug initiation → (Decline 30% or KRT)	Drug initiation → All-cause mortality	(Decline 30% or KRT) → All-cause mortality
**Group**			
H2B	Reference	Reference	Reference
PPI	**1.61(1.44**,** 1.80)**	**3.03(2.10**,** 4.37)**	**1.58(1.24**,** 2.01)**
**Age**,** years**			
**< 65**	Reference	Reference	Reference
**>=65**	**2.11(2.00**,** 2.23)**	**8.08(6.90**,** 9.47)**	**1.17(1.06**,** 1.29)**
**Gender**			
Male	Reference	Reference	Reference
Female	**0.89(0.85**,** 0.92)**	**0.58(0.53**,** 0.63)**	**0.84(0.79**,** 0.90)**
**Gastrointestinal diseases (time-dependent)**			
No	Reference	Reference	Reference
Yes	**1.09(1.03**,** 1.15)**	**0.85(0.75**,** 0.96)**	1.05(0.97, 1.13)
**Cardiovascular and** **cerebrovascular diseases (time-dependent)**			
No	Reference	Reference	Reference
Yes	**2.24(2.11**,** 2.38)**	1.06(0.95, 1.19)	**1.15(1.03**,** 1.28)**
**Diabetes mellitus (time-dependent)**			
No	Reference	Reference	Reference
Yes	**1.56(1.48**,** 1.63)**	**1.09(1.04**,** 1.22)**	0.93(0.86, 1.00)
**Chronic obstructive pulmonary diseases (time-dependent)**			
No	Reference	Reference	Reference
Yes	**1.30(1.24**,** 1.37)**	**1.44(1.30**,** 1.60)**	**1.13(1.05**,** 1.22)**
**NSAIDs/Aspirin (time-dependent)**			
No	Reference	Reference	Reference
Yes	**0.91(0.87**,** 0.95)**	**0.71(0.64**,** 0.79)**	**0.75(0.69**,** 0.82)**
**Statins (time-dependent)**			
No	Reference	Reference	Reference
Yes	**0.76(0.72**,** 0.80)**	**0.42(0.37**,** 0.48)**	**0.70(0.65**,** 0.76)**
**Antithrombotics (time-dependent)**			
No	Reference	Reference	Reference
Yes	**1.40(1.33**,** 1.47)**	**1.44 (1.30**,** 1.60)**	1.04(0.97, 1.12)
**eGFR baseline**			
G1 ( > = 90 mL/min/1.73m^2^)	Reference	Reference	Reference
G2 (60–89 mL/min/1.73m^2^)	**1.35(1.28**,** 1.43)**	1.13(0.99, 1.28)	0.97(0.88, 1.08)
G3A (45–59 mL/min/1.73m^2^)	**2.15(1.99**,** 2.32)**	**1.64(1.39**,** 1.93)**	1.11(0.98, 1.26)
G3B (30–44 mL/min/1.73m^2^)	**2.99(2.75**,** 3.26)**	**2.78(2.33**,** 3.30)**	1.13(0.99, 1.29)
G4 (15–29 mL/min/1.73m^2^)	**4.70(4.20**,** 5.27)**	**4.26(3.42**,** 5.30)**	1.05(0.90, 1.23)

Note: Hazard ratios here represent how the *instantaneous risk* of making a particular transition is modified by the covariate. The lower limit of the hazard ratio (HR) exceeds 1 which suggests an increased risk or hazard rate in the group compared to the reference group. The upper limit of the hazard ratio (HR) is below 1 which indicates a decreased risk or hazard rate in the group compared to the reference group. If the 95% CI includes 1 then it indicates that no significant difference in the hazard rates between the two groups. Boldface values indicate significance.

### Predicting the transition probability over time

A prediction tool called **“**Risk prediction**”** ( https://transitionprobability.shinyapps.io/Risk_Prediction/) was developed based on the derived multistate model. With this tool, we can predict future events that happen to a person who follows this model. For instance, given an individual who is using PPI, a female, older than 65 years old, with an eGFR > = 60 mL/min/1.73m2, without comorbidities of gastrointestinal diseases, cardiovascular/cerebrovascular diseases, diabetes mellitus, COPDs, this person has a 35% probability from “PPI initiate” state to go “All-cause mortality” over 5 years.

## Discussion

In this study, we have proposed a data analytics workflow based on process mining techniques within the context of epidemiology cohort research. Through a detailed case study, we have illustrated the practical application of process mining and its potential contributions to epidemiological research. Process mining can automatically uncover real-world processes, thereby aiding in identifying disease progression patterns that can complement traditional epidemiological methods.

### Process /data-driven methodology

The proposed methodology, with a blended process and data-driven approach, provides a distinctive advantage by offering valuable insights into disease trajectories through the automatic generation of a process map. Traditional epidemiological methods such as survival analysis^7^ typically concentrate on time-to-event analyses with a single endpoint. However, when multiple pivotal events coexist, conventional epidemiological methods become insufficient for comparing diverse trajectories. Process mining, by revealing the underlying structure of events in real-world data, assists in constructing the multistate model. Despite process mining challenges in quantifying intensity and adjusting confounders during comparisons, the multistate model proves crucial for quantifying transitions within the specified context. In fact, the advantages of both models could be combined to provide process–based multistate models, that not only provide a model to quantify the transition within the specified context but also allow to discover the multistate model’s sequences as processes when they are not available. Thus, the proposed methodology allows integrating process mining with a multistate model making it possible to consider multiple events and providing a convenient way to visually represent the time-sequence of the longitudinal processes, facilitating a preliminary understanding of relationships.

### Validating the methodology

We analysed two cohorts (PPI and H2B) and developed two process models with the proposed methodology. By comparing transitions and events between these two groups, we identified substantial differences in the percentual distributions of events *Decline of 30%* and *All-cause mortality*, suggesting potential statistical significance. Subsequently, we conducted multistate modelling to validate the identified transition differences *(Drug initiation → Decline 30%/KRT*, *Drug initiation → All-cause mortality*,* Decline 30%/KRT→ All-cause mortality*). The results indicate that PPI usage is a risk factor for kidney function decline, aligning with previous studies that have highlighted the link between PPI use and adverse kidney outcomes^11,29^. While the mechanisms underlying the association between PPI and renal function impairment are not yet fully understood, various pathways have been proposed, including inhibiting gastric acid secretion, alterations in gut microbiota, and interference with the absorption of essential nutrients such as magnesium^30^. Our analysis revealed several key influencers for each transition (see Table 2). For instance, in the transition from *Drug initiation → Decline 30%/KRT*, our findings identified PPI, older age, gastrointestinal diseases, cardiovascular/cerebrovascular diseases, diabetes mellitus, and chronic obstructive pulmonary diseases as risk factors, which align with previous publications^31–35^.

### Clinical implications

The higher risk of kidney function decline associated with PPI usage, as demonstrated by a more than 30% decrease in eGFR, points to the need for cautious prescribing practices. Clinicians should consider the risks of PPI in patients with compromised kidney function and evaluate the necessity and duration of PPI therapy in these individuals. Our study also supports the potential benefits of opting for H2B over PPI in managing conditions necessitating acid suppression, especially in patients at risk of chronic kidney disease progression.

### Prediction from the proposed methodology

The conceptualisation of the CKD progression process, as revealed through the process and multistate modelling, highlights an evolving trajectory over time. This model serves as an important tool for predicting the future disease prognosis of an individual based on their baseline information. It is important to emphasise that the current predictive model relies on real-world clinical data. Envision the transformative potential of our predictive model if our dataset were more comprehensive, particularly in its ability to provide valuable clinical applications. This holds especially true for patients with end-stage renal disease, facilitating periodic monitoring and strategic planning for dialysis interventions.

### Insights from the real-world data through the proposed methodology

The visualisation of disease progression trajectories, as demonstrated in our proposed methodology, can yield significant insights by elucidating relationships between different stages of disease progression. In this context, process mining, applied to time-stamped data such as cohort studies or electronic health records (EHRs), emerges as a potent tool. A study in this field investigated the progression trajectories of Amyotrophic Lateral Sclerosis (ALS) utilising process mining techniques^36^. This research focused on deciphering ALS’s progression mechanisms. The goal was to enhance the prediction of ALS prognosis, thereby improving the quality of life for patients and providing clinicians with valuable insights for treatment planning. Furthermore, process mining has shown potential in modelling disease patterns over time, as suggested by Kusuma^37^. By extracting diagnosis codes from EHRs, this method offers a comprehensive suite of tools for mining care pathways and extracting event data from EHRs. These insights are useful in understanding disease trajectories, especially in chronic conditions. There is a growing literature body generating disease trajectories using EHR data. With the extensive patient data available in EHRs, there is a rich resource for mapping out disease diagnoses over time. The process mining of this data, as highlighted by Kusuma^38^, assists in identifying patterns in disease trajectories, potentially contributing to a clearer understanding of disease progression. In essence, the trajectory of disease progression, when visualised and analysed through process mining techniques, can reveal intricate relationships between different disease stages. This approach, leveraging the wealth of data in EHRs, continues to be a crucial factor in advancing our understanding of chronic diseases and enhancing patient care.

### Limitations, challenges and future work in analysing process with real world data

Process mining can serve as a valuable tool in the investigation of longitudinal processes, yet its application may introduce complexities in research design. The intricate and dynamic nature of real-world processes poses significant challenges in comparing groups that lack homogeneity at baseline. A significant challenge arises when comparing groups with substantial disparities in baseline characteristics, such as those observed in our case study between new users of PPI and H2B. These groups exhibited significant differences in baseline attributes such as age, sex proportion, comorbidity prevalence, and the frequency of concomitant medication dispensation. Such heterogeneity underscores the potential for confounding and bias inherent in observational study designs. Despite employing a sophisticated multistate model to adjust for known confounders, residual confounding from unmeasured or unknown factors could still influence our results.

Furthermore, the reliance on administrative data and prescription records may not accurately reflect over-the-counter medication usage, such as intermittent PPI use, which could lead to misclassification of exposure. Additionally, the study’s inclusion criteria restricted participation to new users of PPI and H2B who had a baseline kidney function test recorded. These criteria may exclude patients with less healthcare engagement, who might exhibit different risks.

Although process mining tools enhance data navigability and facilitate a deeper understanding of process behaviours, extracting meaningful insights from complex processes often requires domain-specific knowledge. In this case study, the involvement of epidemiologist co-authors was instrumental. Their expert insights led to crucial refinements in the initial process model, particularly in the abstraction of events. They identified potential overlaps in events, such as between a 30% decline in kidney function and end-stage renal disease. They recommended excluding the end-stage renal disease event to clarify disease progression and prevent event overlap. This underscores the importance of conducting process analysis interactively, engaging domain experts in the ‘data rodeos’^39^. The involvement of domain experts and an interactive approach^39^ enhances the validity and interpretability of process mining outcomes, ensuring analysis is informed and contextually appropriate.

Applying this methodology to broader research questions in longitudinal epidemiology and extending process mining to other diseases are promising future research avenues. Utilising the visualisation strengths of process mining could automate the identification of relationships among diseases and medications, advancing the development of data-driven hypotheses.

## Conclusion

In conclusion, this study proposes a methodology for applying process mining in epidemiological studies, as demonstrated through a case study. The case study served to showcase the application of process mining and the generation of valuable insights from data. We showed the potential of process mining as a useful tool for unfolding the underlying processes and patterns of events in epidemiological cohort research. It facilitates an in-depth examination of disease progression patterns and the extraction of insights from data.

## Electronic supplementary material

Below is the link to the electronic supplementary material.

Supplementary Material 1

## Data Availability

The SCREAM contains sensitive personal data that cannot be publicly shared due to GDPR regulations. We welcome collaboration project proposals that adhere to GDPR, national, and institutional regulations concerning data sharing and access. For inquiries, please contact juan.jesus.carrero@ki.se.

## References

[1] Maruster, L., van der Aalst, W., Weijters, T., van den Bosch, A. & Daelemans, W. Automated discovery of workflow models from hospital data. *B Kr€ Oose M De Rijke* **18** (2001).

[2] Rojas, E., Munoz-Gama, J., Sepúlveda, M. & Capurro, D. Process mining in Healthcare: A literature review. *J. Biomed. Inf.*10.1016/j.jbi.2016.04.007 (2016). 10.1016/j.jbi.2016.04.007 27109932

[3] Cuendet, M. A. et al. A differential process mining analysis of COVID-19 management for cancer patients. *Front. Oncol.* **12**, 1043675. 10.3389/fonc.2022.1043675 (2022). 36568192 10.3389/fonc.2022.1043675 PMC9768429

[4] Zhang, Y. & Padman, R. Innovations in chronic care delivery using data-driven clinical pathways. *Am. J. Manag. Care*. **21**, e661–e668 (2015). 26760429

[5] Chen, K., Abtahi, F., Carrero, J. J., Fernandez-Llatas, C. & Seoane, F. Process mining and data mining applications in the domain of chronic diseases: a systematic review. *Artif. Intell. Med.* **144**, 102645. 10.1016/j.artmed.2023.102645 (2023). 37783545 10.1016/j.artmed.2023.102645

[6] Twisk, J. W. R. & de Vente, W. Hybrid models were found to be very elegant to disentangle longitudinal within- and between-subject relationships. *J. Clin. Epidemiol.* **107**, 66–70. 10.1016/j.jclinepi.2018.11.021 (2019). 30500406 10.1016/j.jclinepi.2018.11.021

[7] Kleinbaum, D. G. & Klein, M. *Survival Analysis a self-learning text* (Springer, 1996).

[8] Makunts, T., Cohen, I. V., Awdishu, L. & Abagyan, R. Analysis of postmarketing safety data for proton-pump inhibitors reveals increased propensity for renal injury, electrolyte abnormalities, and nephrolithiasis. *Sci. Rep.* **9** (2019). 10.1038/s41598-019-39335-7 PMC6381091 30783195

[9] Morschel, C. F., Mafra, D. & Eduardo, J. C. C. The relationship between proton pump inhibitors and renal disease. *Jornal Brasileiro De Nefrologia*. **40**, 301–306 (2018). 30010692 10.1590/2175-8239-JBN-2018-0021 PMC6533960

[10] Al-Aly, Z., Maddukuri, G. & Xie, Y. Proton Pump inhibitors and the kidney: implications of current evidence for clinical practice and when and how to Deprescribe. *Am. J. Kidney Diseases: Official J. Natl. Kidney Foundation* (2020). 10.1053/j.ajkd.2019.07.012 31606235

[11] Klatte, D. C. F. et al. Association between Proton pump inhibitor use and risk of progression of chronic kidney disease. *Gastroenterology*. **153**, 702–710. 10.1053/j.gastro.2017.05.046 (2017). 28583827 10.1053/j.gastro.2017.05.046

[12] Runesson, B. et al. The Stockholm CREAtinine measurements (SCREAM) project: protocol overview and regional representativeness. *Clin. Kidney J.* **9**, 119–127. 10.1093/ckj/sfv117 (2016). 26798472 10.1093/ckj/sfv117 PMC4720196

[13] Levey, A. S. et al. A New equation to Estimate glomerular filtration rate. *Ann. Intern. Med.* **150**, 604. 10.7326/0003-4819-150-9-200905050-00006 (2009). 19414839 10.7326/0003-4819-150-9-200905050-00006 PMC2763564

[14] Chap. 1: Definition and classification of CKD. *Kidney International Supplements* 3, 19–62, doi: (2013). 10.1038/kisup.2012.64 10.1038/kisup.2012.64 PMC4089693 25018975

[15] Janssenswillen, G. et al. Enabling reproducible business process analysis. *Knowl. Based Syst.* **163**, 927–930. 10.1016/j.knosys.2018.10.018 (2019).

[16] Gatta, R. et al. in *Artificial Intelligence in Medicine.* (eds Annette ten Teije, Christian Popow, John H. Holmes, & Lucia Sacchi) 351–355 (Springer International Publishing). 10.1016/j.artmed.2018.10.005 30409394

[17] Valero-Ramon, Z., Fernandez-Llatas, C., Valdivieso, B. & Traver, V. Dynamic models supporting personalised chronic Disease Management through Healthcare Sensors with interactive process mining. *Sens. (Basel)*. **20**10.3390/s20185330 (2020). 10.3390/s20185330 PMC7570892 32957673

[18] Van Dongen, B. F., de Medeiros, A. K. A., Verbeek, H., Weijters, A. & van Der Aalst, W. M. in *Applications and Theory of Petri Nets* : 26th International Conference, ICATPN 2005, Miami, USA, June 20–25, 2005. Proceedings 26. 444–454 (Springer). (2005).

[19] Fernandez-Llatas, C., Lizondo, A., Monton, E. & Benedí, J. M. Process mining methodology for health process tracking using real-time indoor location systems. *Sensors*. 10.3390/s151229769 (2015). 26633395 10.3390/s151229769 PMC4721690

[20] Fernandez-Llatas, C. in *Interactive Process Mining in Healthcare* (ed Carlos Fernandez-Llatas) 141–162Springer International Publishing, (2021).

[21] Fani Sani, M., van Zelst, S. J. & van der Aalst, W. M. P. The impact of biased sampling of event logs on the performance of process discovery. *Computing*. **103**, 1085–1104. 10.1007/s00607-021-00910-4 (2021).

[22] De Koninck, P., De Weerdt, J. & vanden Broucke, S. K. L. M. explaining clusterings of process instances. *Data Min. Knowl. Disc.* **31**, 774–808. 10.1007/s10618-016-0488-4 (2017).

[23] Pasquadibisceglie, V., Appice, A., Castellano, G. & van der Aalst, W. Coupling predictive process mining to process discovery. *Inform. Sci. 606*. **PROMISE**, 250–271. 10.1016/j.ins.2022.05.052 (2022).

[24] Diba, K., Batoulis, K., Weidlich, M. & Weske, M. Extraction, correlation, and abstraction of event data for process mining. *WIREs Data Min. Knowl. Discov.* **10**10.1002/widm.1346 (2019).

[25] Inker, L. A. et al. KDOQI US commentary on the 2012 KDIGO clinical practice guideline for the evaluation and management of CKD. *Am. J. Kidney Dis.* **63**, 713–735. 10.1053/j.ajkd.2014.01.416 (2014). 24647050 10.1053/j.ajkd.2014.01.416

[26] Lintu, M. K., Shreyas, K. M. & Kamath, A. A multi-state model for kidney disease progression. *Clin. Epidemiol. Global Health*. **13**10.1016/j.cegh.2021.100946 (2022).

[27] Grover, G., Sabharwal, A., Kumar, S. & Thakur, A. K. A multi-state Markov Model for the progression of chronic kidney disease. *Turkiye Klinikleri J. Biostatistics* **11** (2019).

[28] Jackson, C. Multi-state models for Panel Data: the Msm Package for R. *J. Stat. Softw.* **38**, 1–28. 10.18637/jss.v038.i08 (2011).

[29] Paueksakon, P. & Fogo, A. B. Do Proton-Pump Inhibitors Cause CKD and Progression of CKD? COMMENTARY. *Kidney360* 3, 1141–1143, doi: (2022). 10.34067/KID.0008302021 10.34067/KID.0008302021 PMC9337904 35920527

[30] Al-Aly, Z., Maddukuri, G. & Xie, Y. Proton Pump inhibitors and the kidney: implications of current evidence for clinical practice and when and how to Deprescribe. *Am. J. Kidney Dis.*10.1053/j.ajkd.2019.07.012 (2020). 31606235 10.1053/j.ajkd.2019.07.012

[31] Kitai, Y., Nangaku, M. & Yanagita, M. Aging-related kidney diseases. *Contrib. Nephrol.* **199**, 1–8 (2021). 34343996 10.1159/000517708

[32] Chang, P. Y. et al. Risk factors of gender for renal progression in patients with early chronic kidney disease. *Med. (Baltim).* 95 (2016). 10.1097/MD.0000000000004203 PMC5265827 27472690

[33] Anavekar, N. S. & Pfeffer, M. A. Cardiovascular risk in chronic kidney disease. *Kidney Int. Suppl.* **92**, S11–15 (2004). 10.1111/j.1523-1755.2004.09203.x 15485401

[34] Jung, H. H. Evaluation of serum glucose and kidney disease progression among patients with diabetes. *JAMA Netw. Open.* **4** (2021). 10.1001/jamanetworkopen.2021.27387 PMC8482057 34586368

[35] Park, S. et al. Kidney function and obstructive lung disease: a bidirectional mendelian randomisation study. *Eur. Respir. J.* **58** (2021). 10.1183/13993003.00848-2021 33958431

[36] Tavazzi, E. et al. Leveraging process mining for modeling progression trajectories in amyotrophic lateral sclerosis. *BMC Med. Inf. Decis. Mak.* **22**, 346. 10.1186/s12911-023-02113-7 (2023). 10.1186/s12911-023-02113-7 PMC9896660 36732801

[37] Kusuma, G. P. et al. Process mining of Disease trajectories: a Literature Review. *Stud. Health Technol. Inf.* **281**, 457–461. 10.3233/SHTI210200 (2021). 10.3233/SHTI210200 34042785

[38] Kusuma, G. et al. Springer International Publishing,. in *Lecture Notes in Business Information Processing* 305–316 (2021).

[39] Fernandez-Llatas, C. in *Interactive Process Mining in Healthcare* (ed Carlos Fernandez-Llatas) 1–9Springer International Publishing, (2021).

