# Supplementary material for: Validation of an interactive process mining methodology for clinical epidemiology through a cohort study on chronic kidney disease progression
Source: Sci Rep. 2024 Nov 14;14:27997. doi: 10.1038/s41598-024-79704-5 (PMC11564739; doi:10.1038/s41598-024-79704-5)
Supplement: Supplementary file 1 — Supplementary Material 1 [file 41598_2024_79704_MOESM1_ESM.docx]

Supplementary Table 1 The ICD-10 codes of comorbidities and ATC codes of PPI/H2B

| **Comorbidities** | **ICD-10 codes** |
| --- | --- |
| Gastroesophageal reflux disease | K21 |
| Upper gastrointestinal tract bleeding | K922 |
| Ulcer disease | K221, K25, K26, K27, K28 |
| H. Pylori infection | B980 |
| Myocardial infarction | I21, I22, I252 |
| Cerebrovascular disease | G45-46, H340, I60-69 |
| Peripheral vascular disease | I70, I71, I731, I738, I739, I771, I790, I792, K551, K558, K559, Z958, Z959 |
| Congestive heart failure | I099, I110, I130, I132, I255, I420, I425-429, I43, I50, P290 |
| Hypertension | I10-15 |
| Diabetes mellitus | E10-14 |
| Chronic obstructive pulmonary disease | I278, I279, J40-47, J60-67, J684, J701, J703 |
| **PPI/H2B** | **ATC codes** |
| Proton pump inhibitors | A02BC |
| H2 blockers | A02BA |
| **Concomitant medication** | **ATC codes** |
| NSAIDs, aspirin | M01A |
| Statins | C10AA |
| Antithrombotics | B01A |


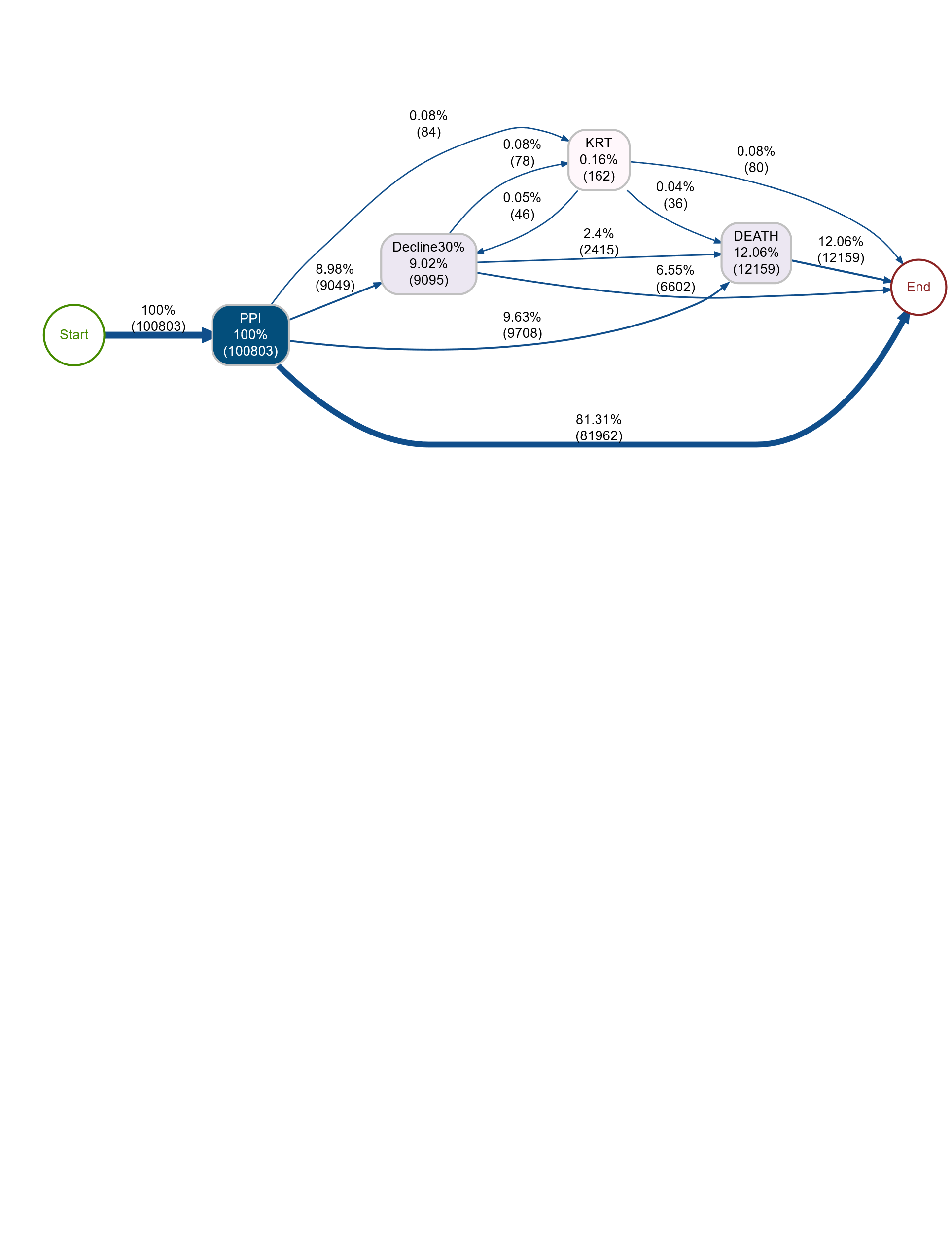

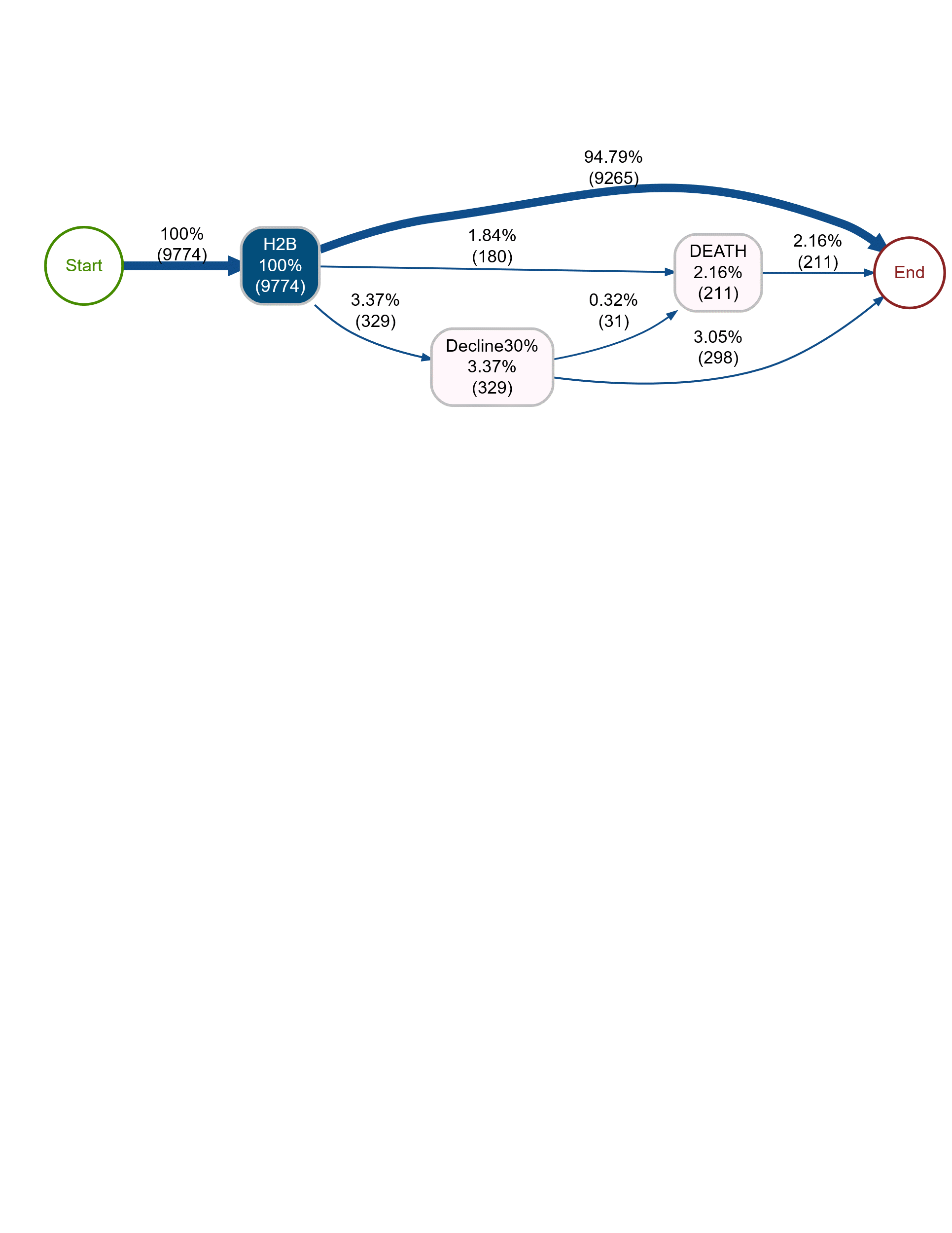


**Supplementary Figure 1.**  Interactive process indicators generated from “bupaR”; the nodes are the events/states with darker color means the more events frequency; Arrows represent time-ordered sequences of traces; the thickness of these arrows corresponds to the frequency of occurrence.; the definition of each node are: "Drug Initiate" - start date for Proton Pump Inhibitors (PPI) or Histamine-2 Blockers (H2B); "Decline30%" - 30% or more reduction in baseline kidney function (eGFR); "KRT" - Kidney Replacement Therapy (includes transplant and dialysis, as per Swedish Renal Registry); "Death" - all-cause mortality.

**
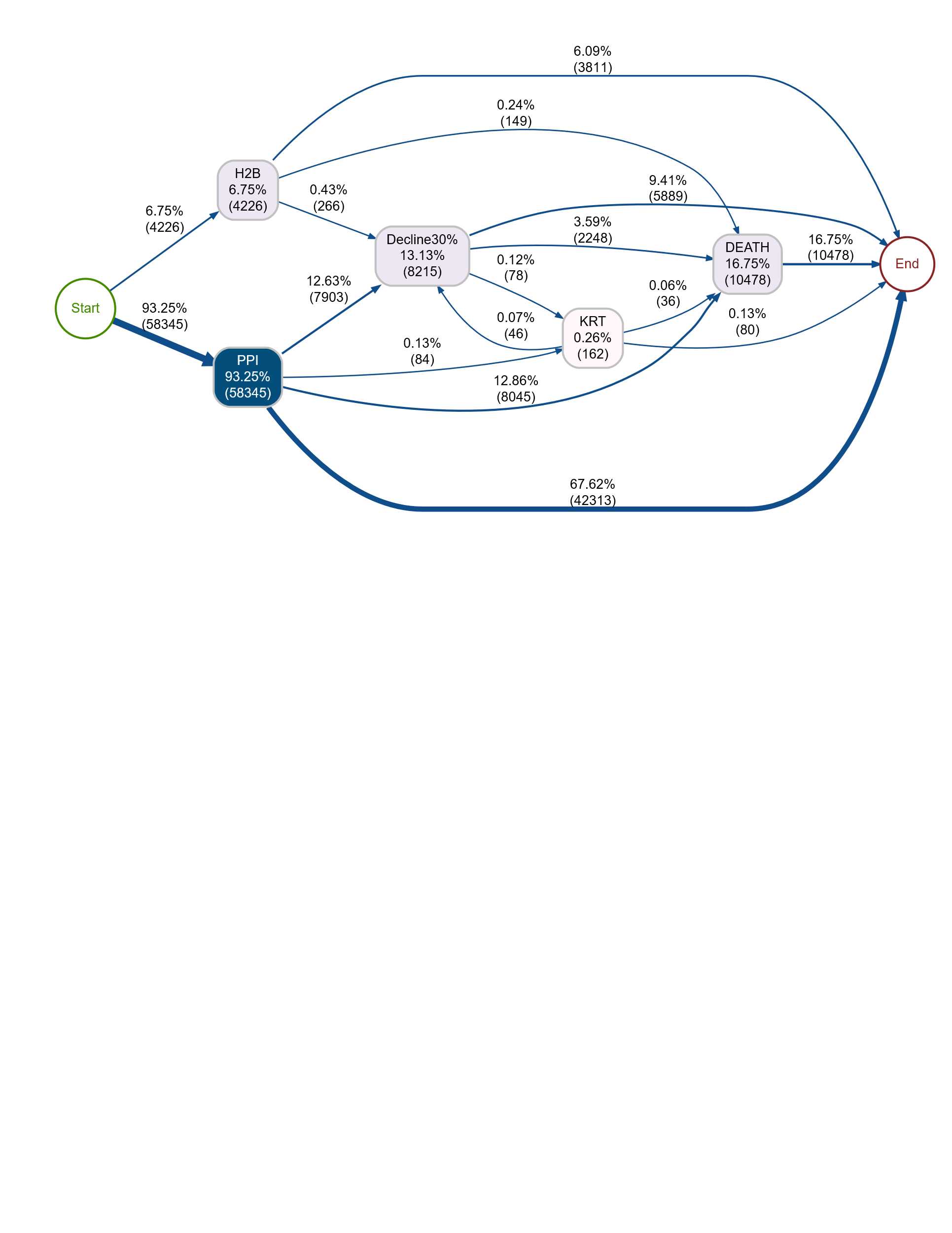
*
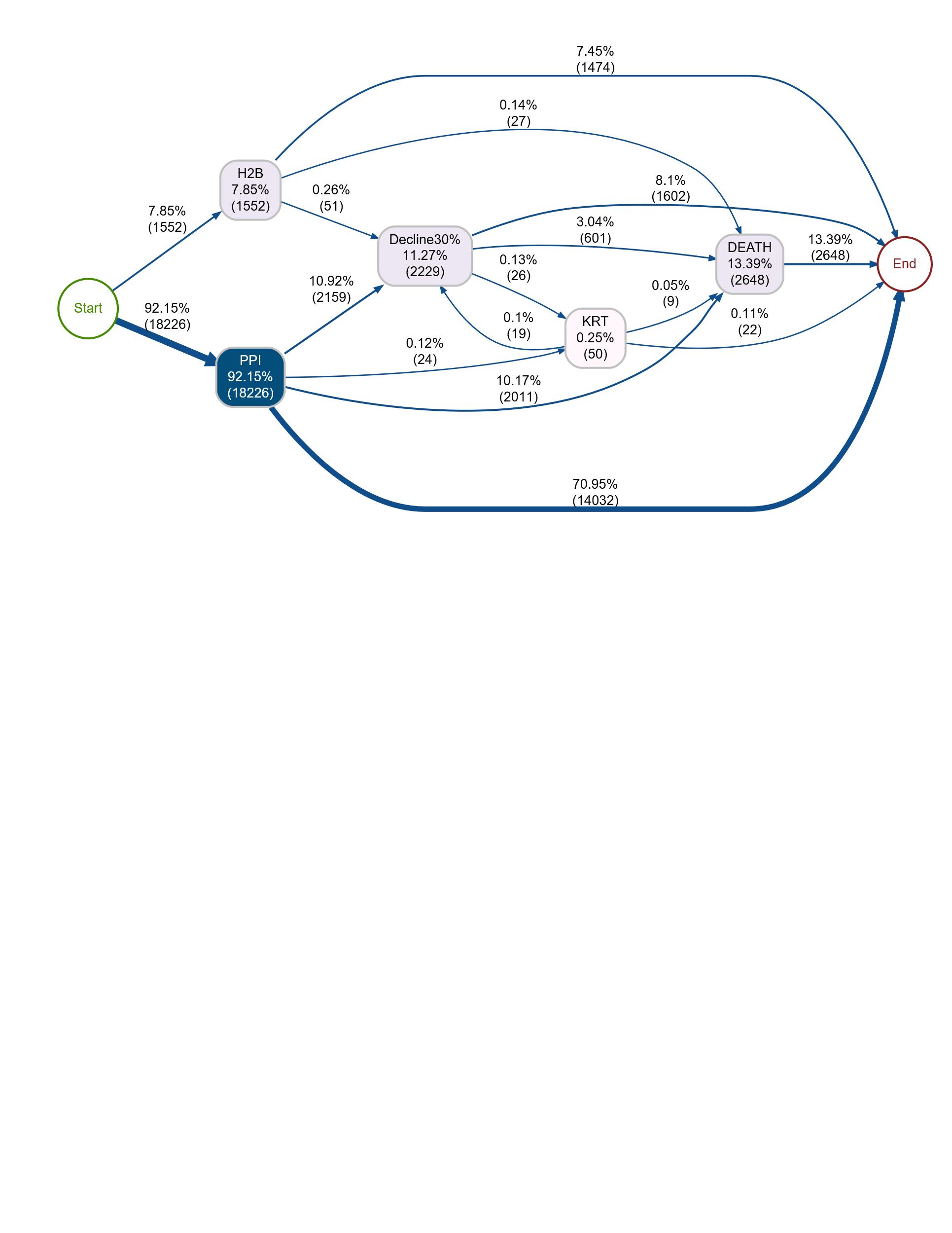
*
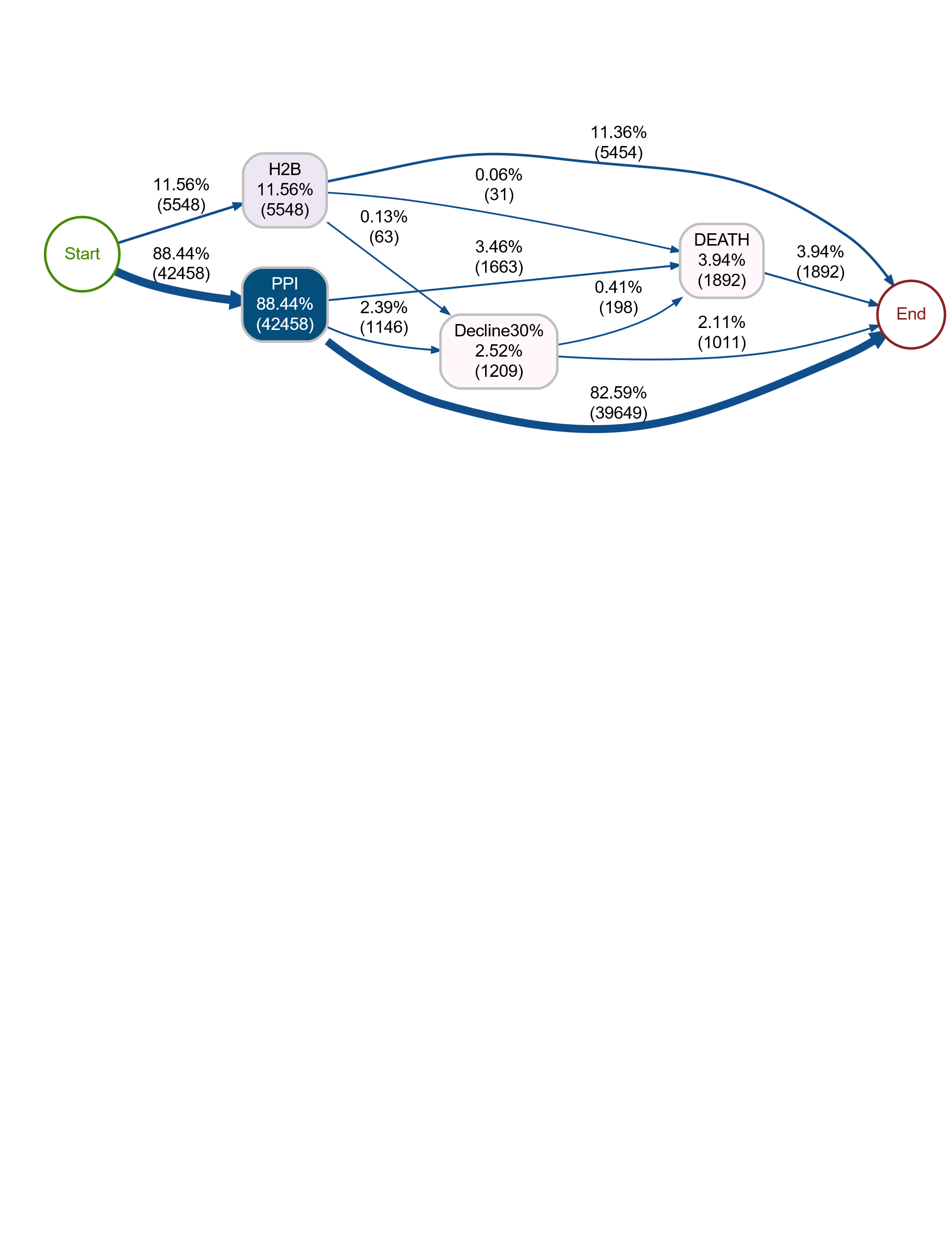
*
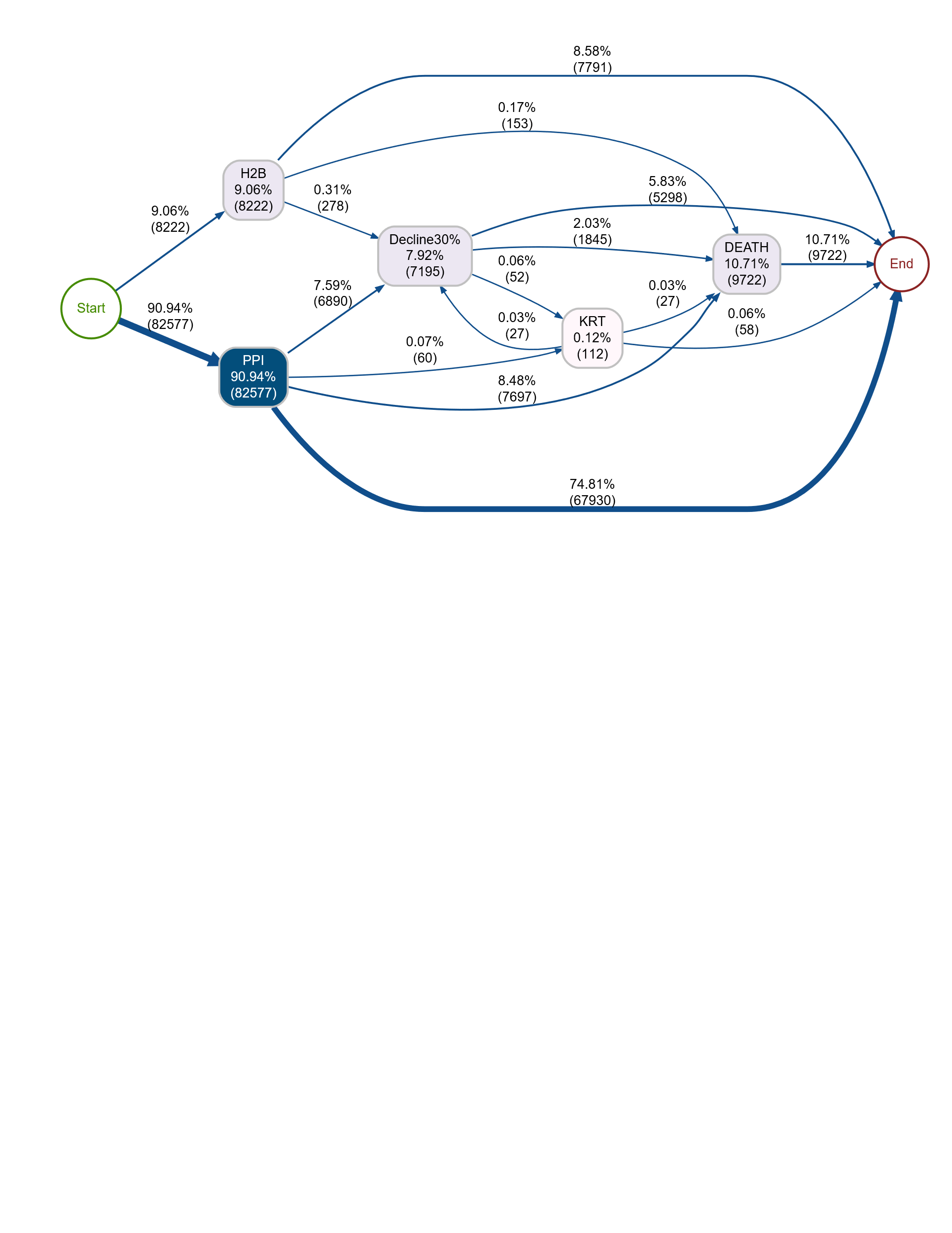
***

Without Cardiovascular/Cerebrovascular disease

With Cardiovascular/Cerebrovascular disease

***Supplementary Figure 3. Filtered Process indicators for people with and without Cardiovascular/Cerebrovascular diseases***

Without Gastrointestinal disease

With Gastrointestinal disease

***Supplementary Figure 2. Filtered Process indicators for people with and without Gastrointestinal diseases***

Without Diabetes

With Diabetes

***Supplementary Figure 4. Filtered Process indicators for people with and without diabetes***


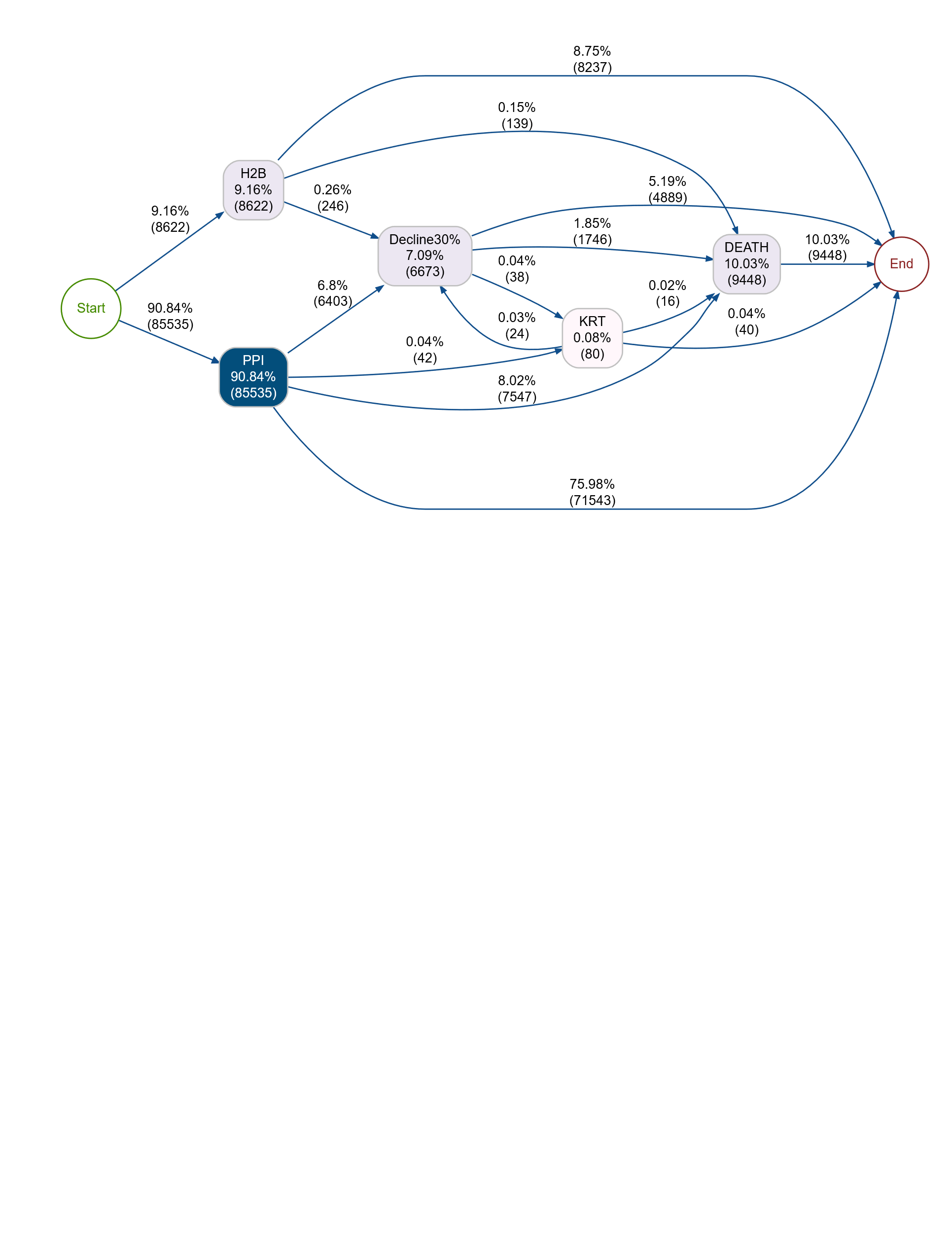

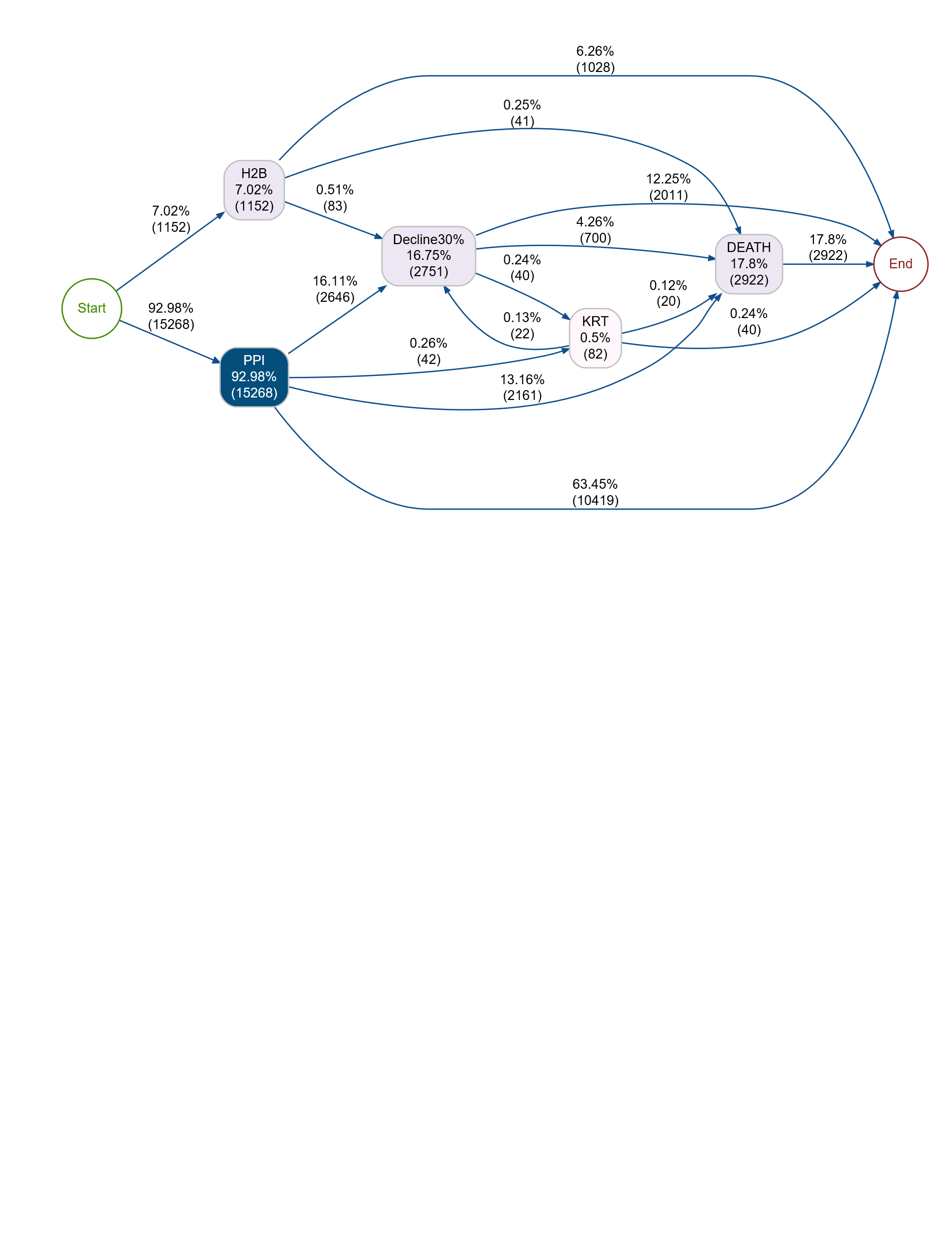


Without COPD

With COPD

***Supplementary Figure 5. Filtered Process indicators for people with and without COPD***


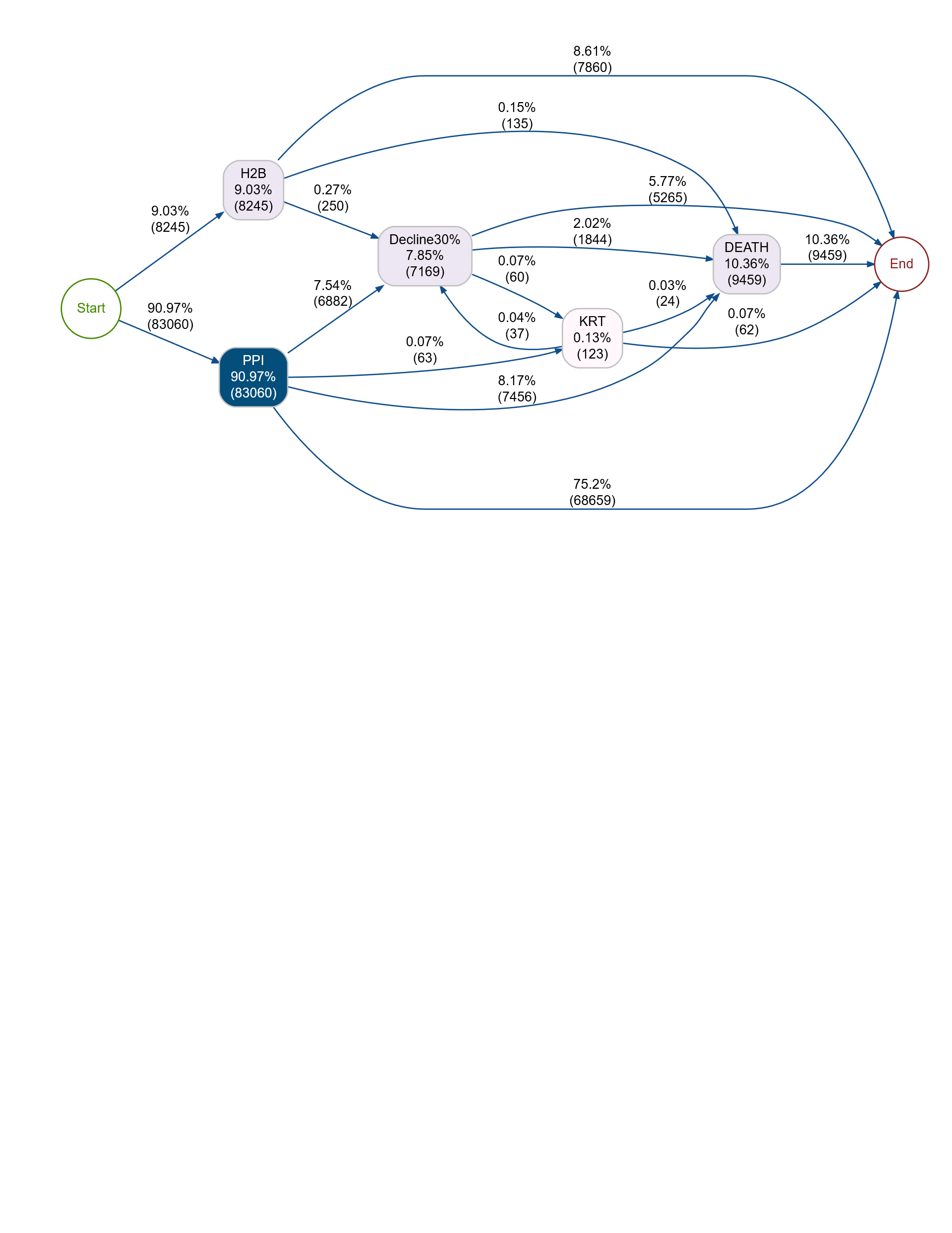

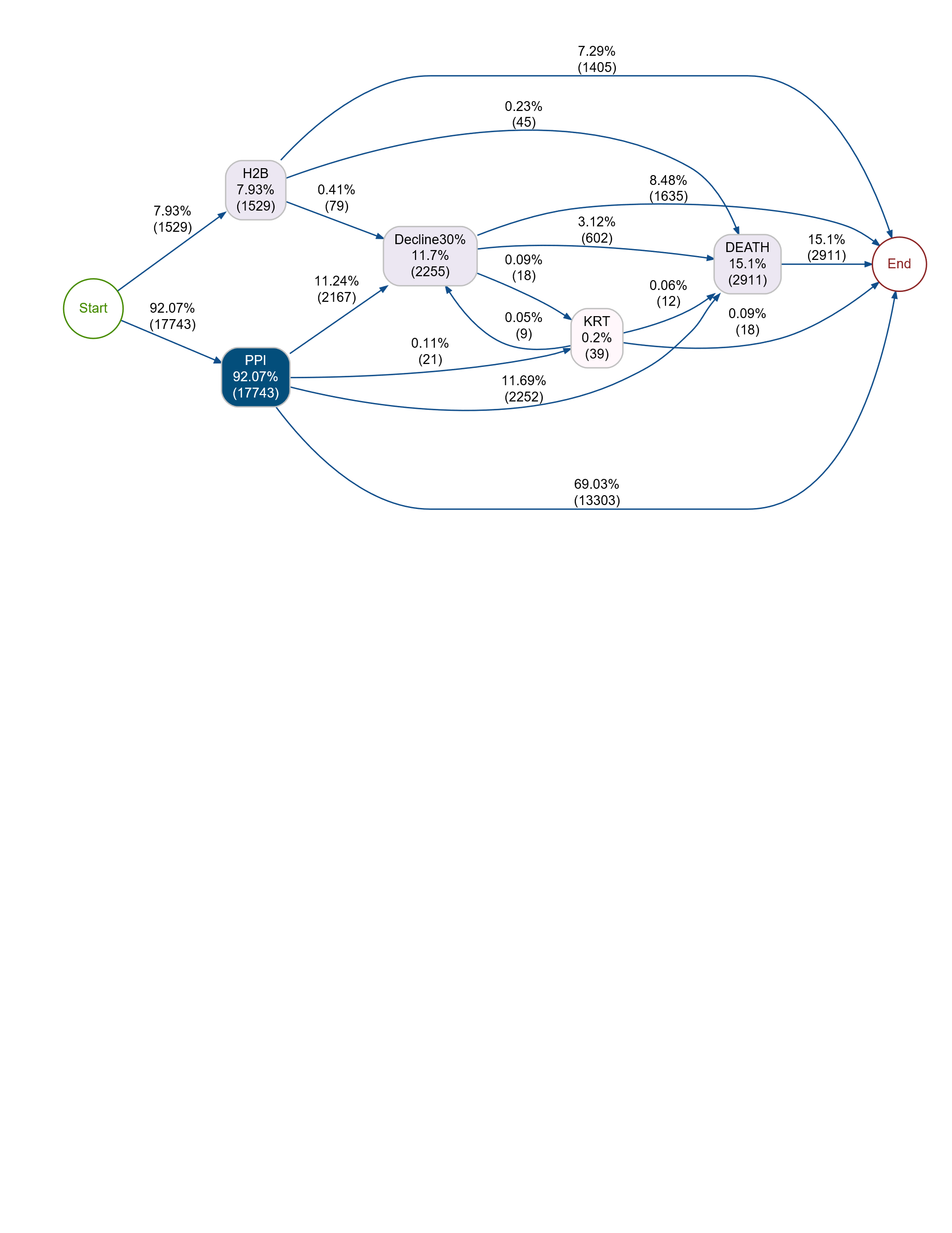


Age <65 years

Age >=65 years

***Supplementary Figure 6. Filtered Process indicators for age***


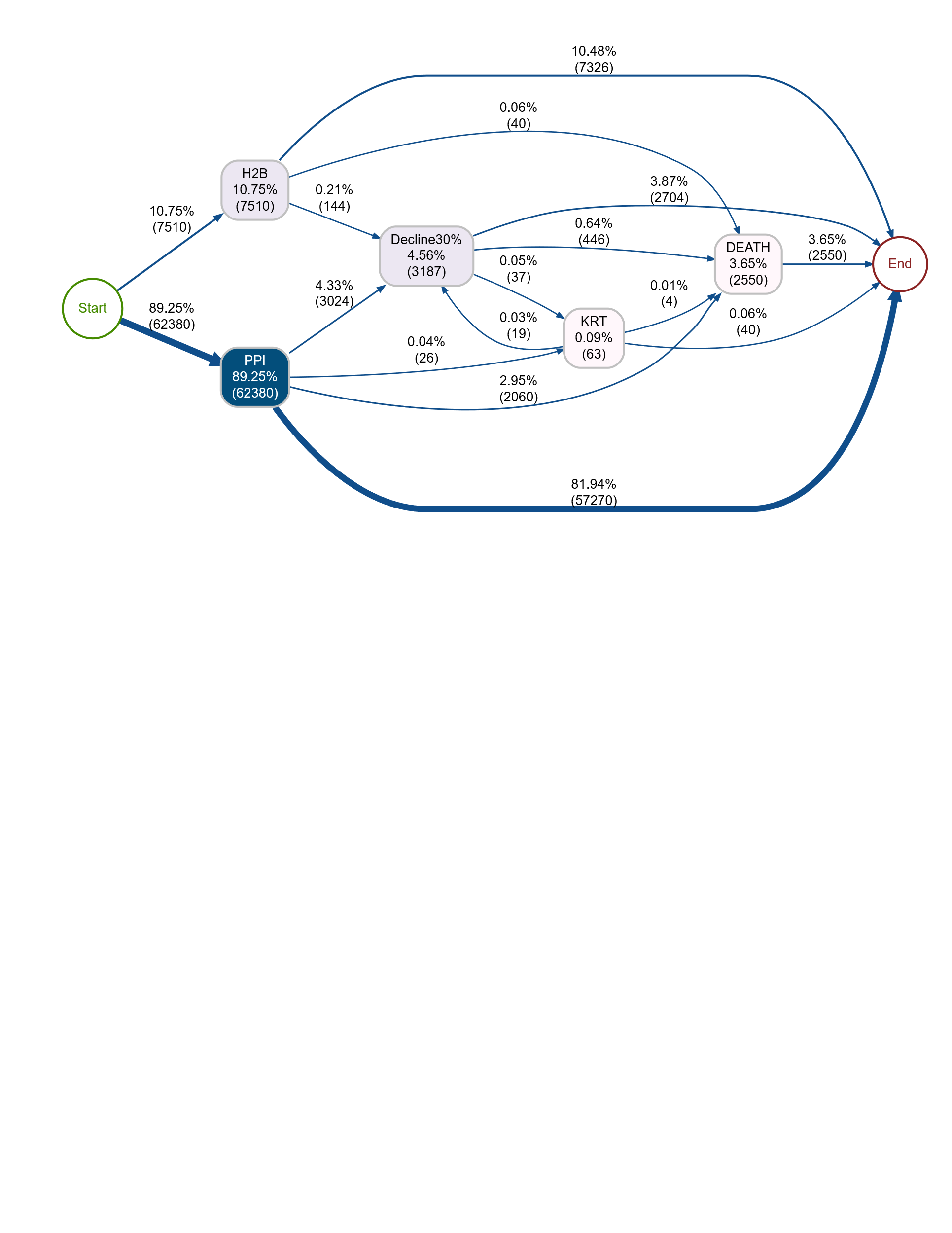

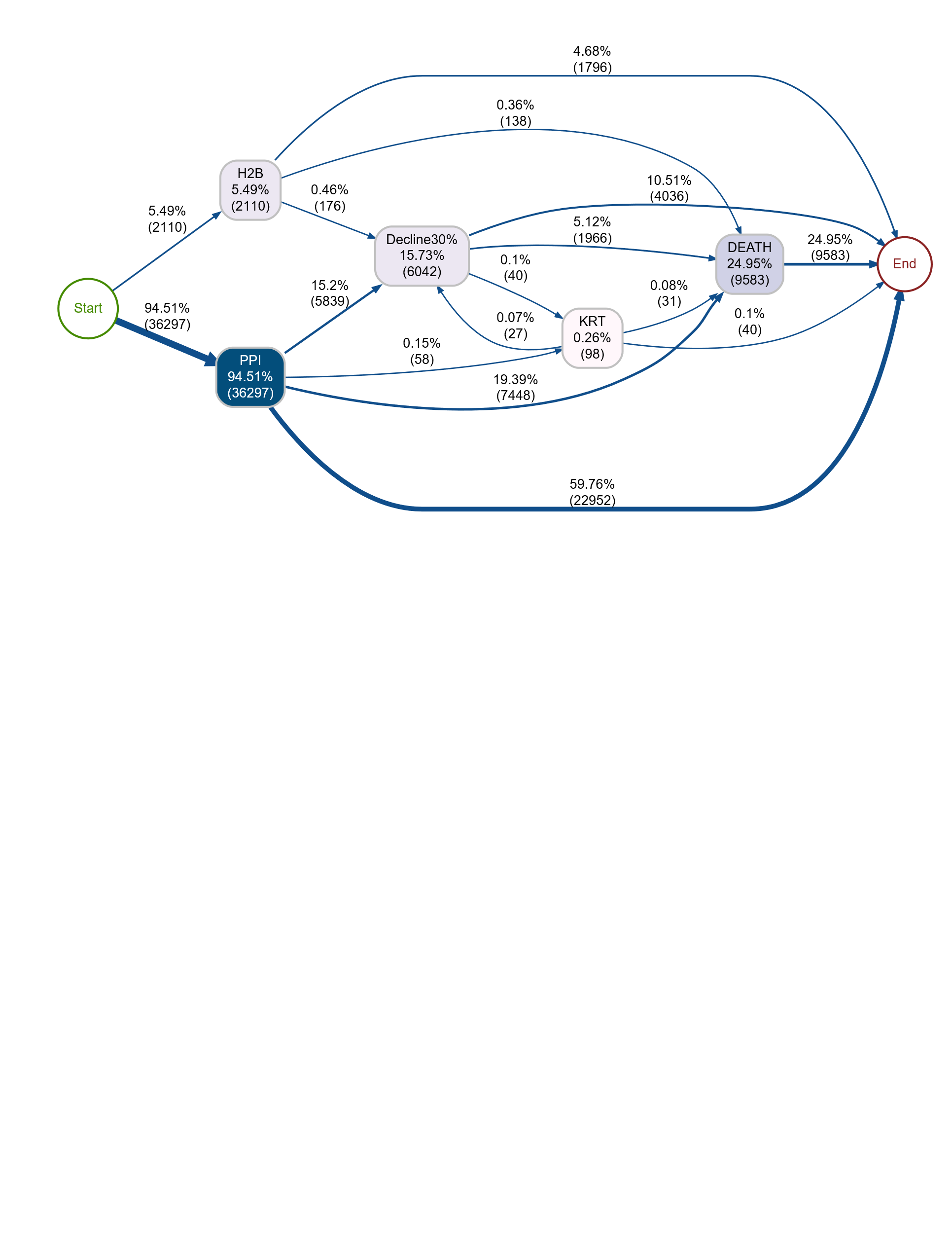


**
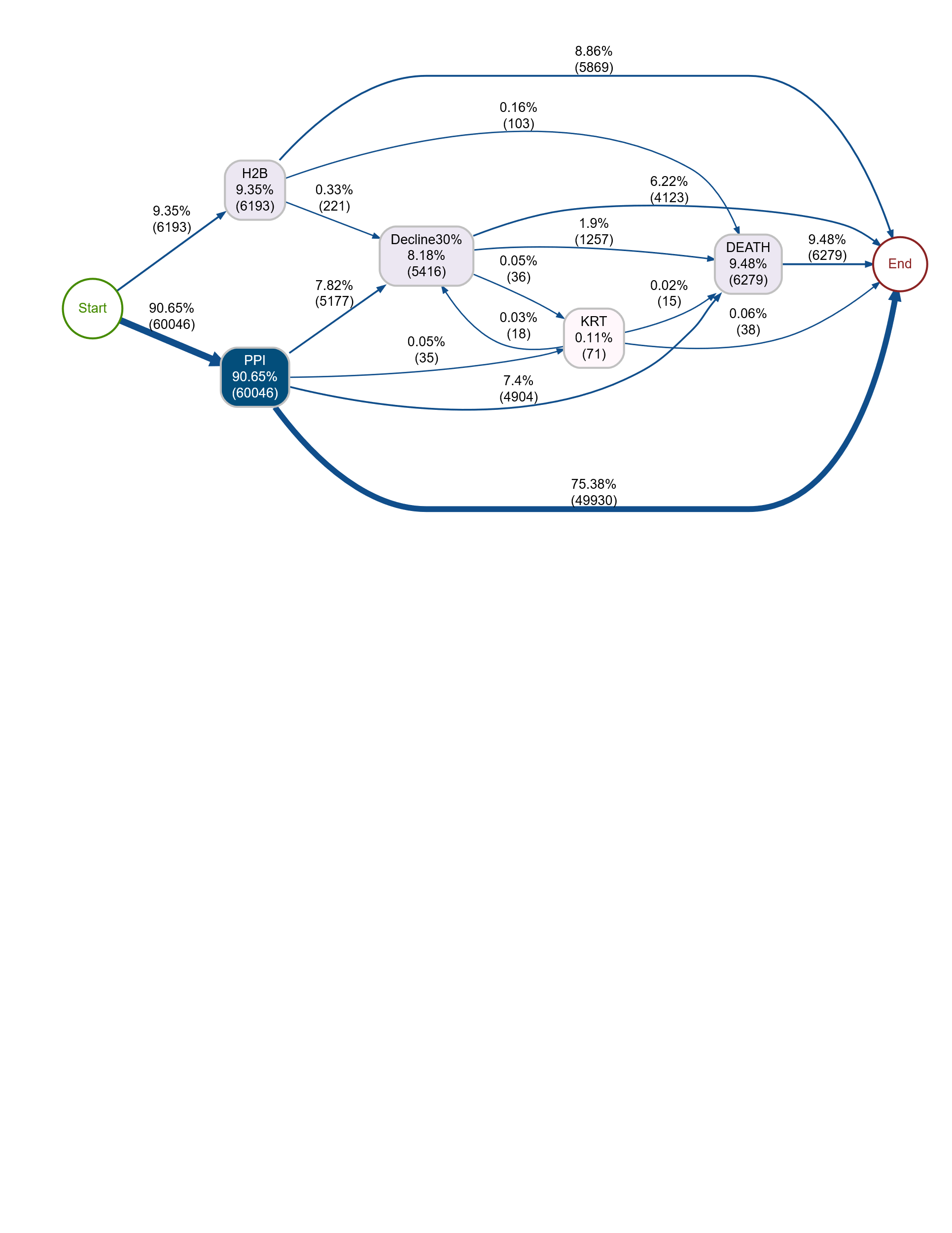

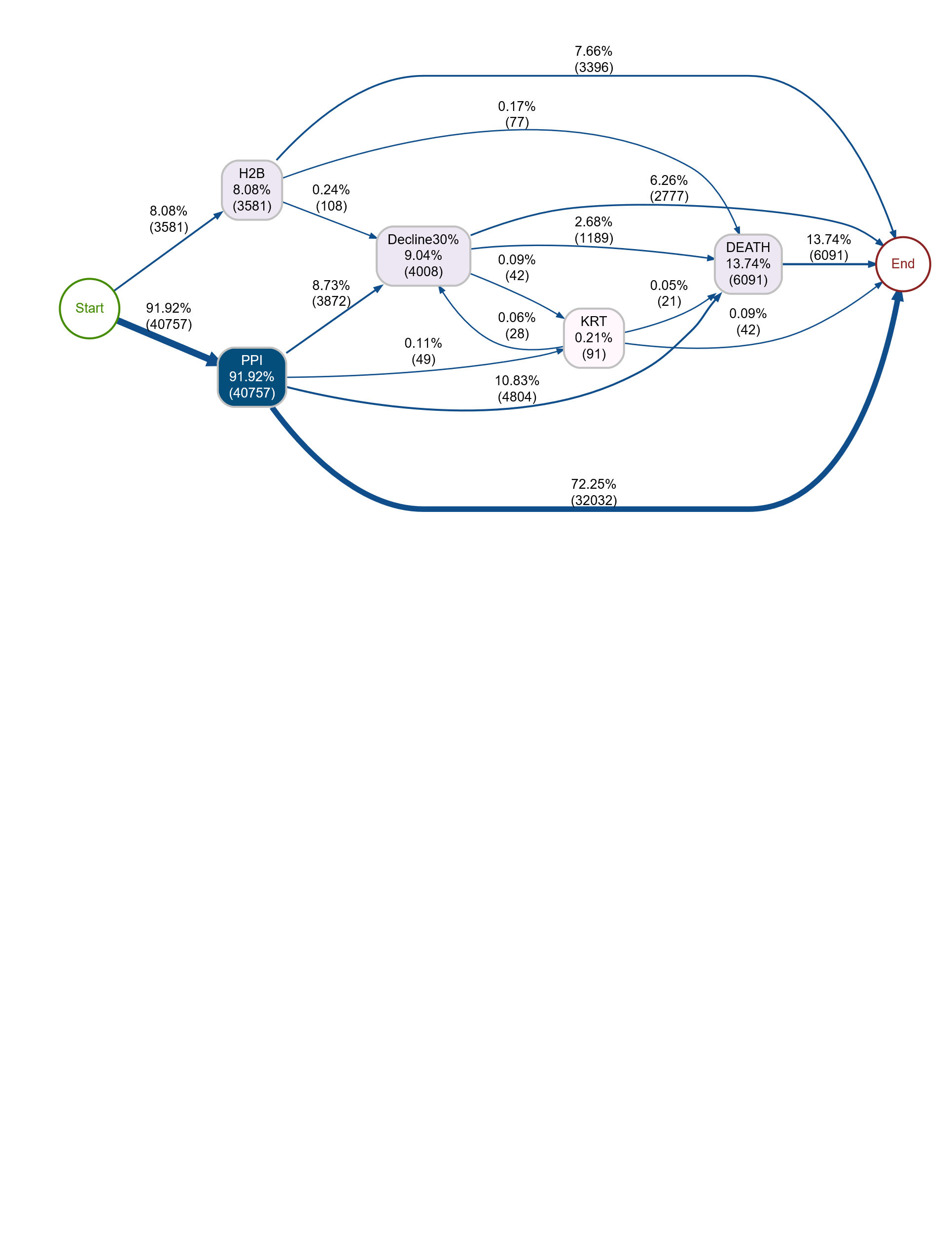
**

Male

Female

***Supplementary Figure 7. Filtered Process indicators for gender***

eGFR 1

eGFR 2

***Supplementary Figure 8. Filtered Process indicators for baseline eGFR***

**
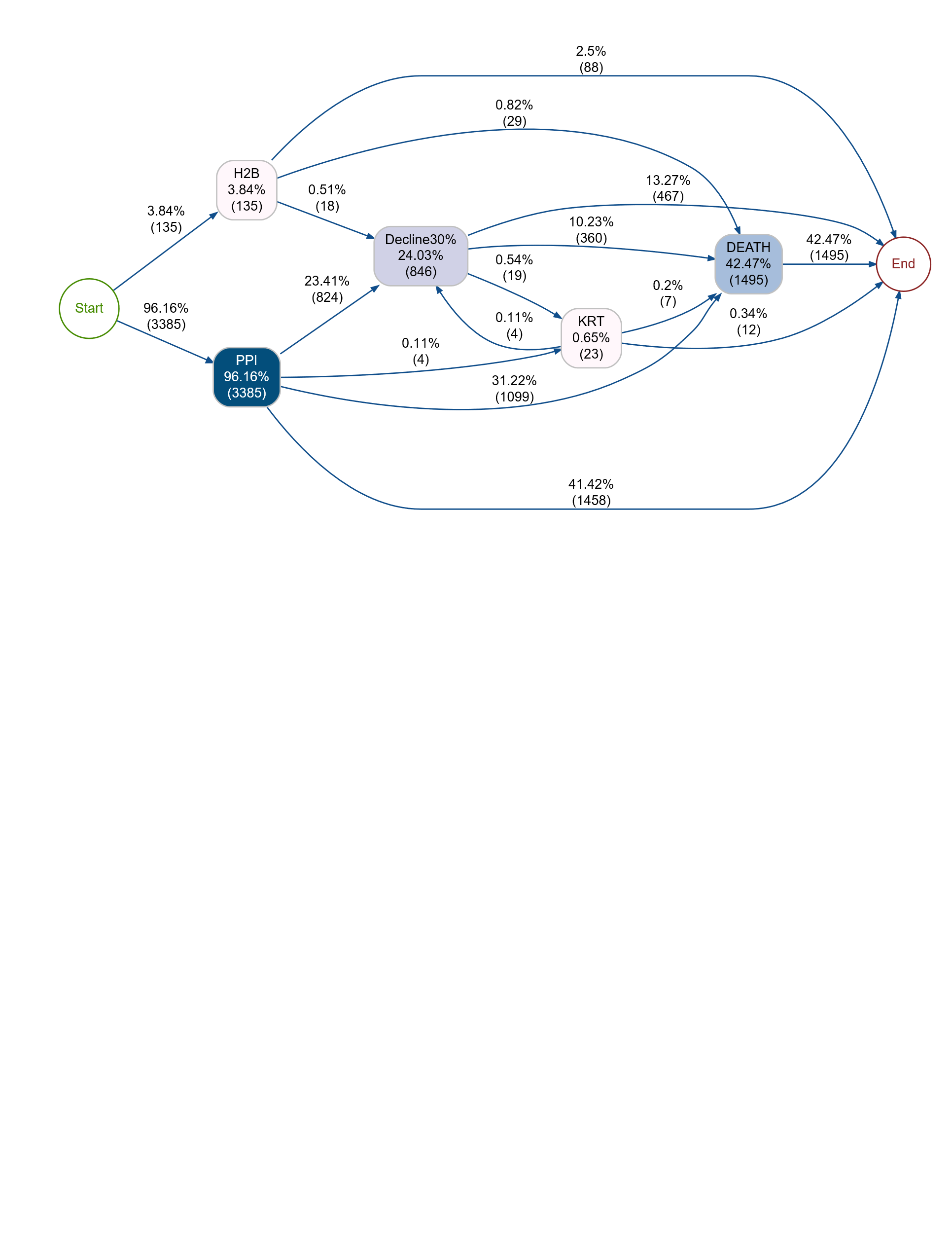

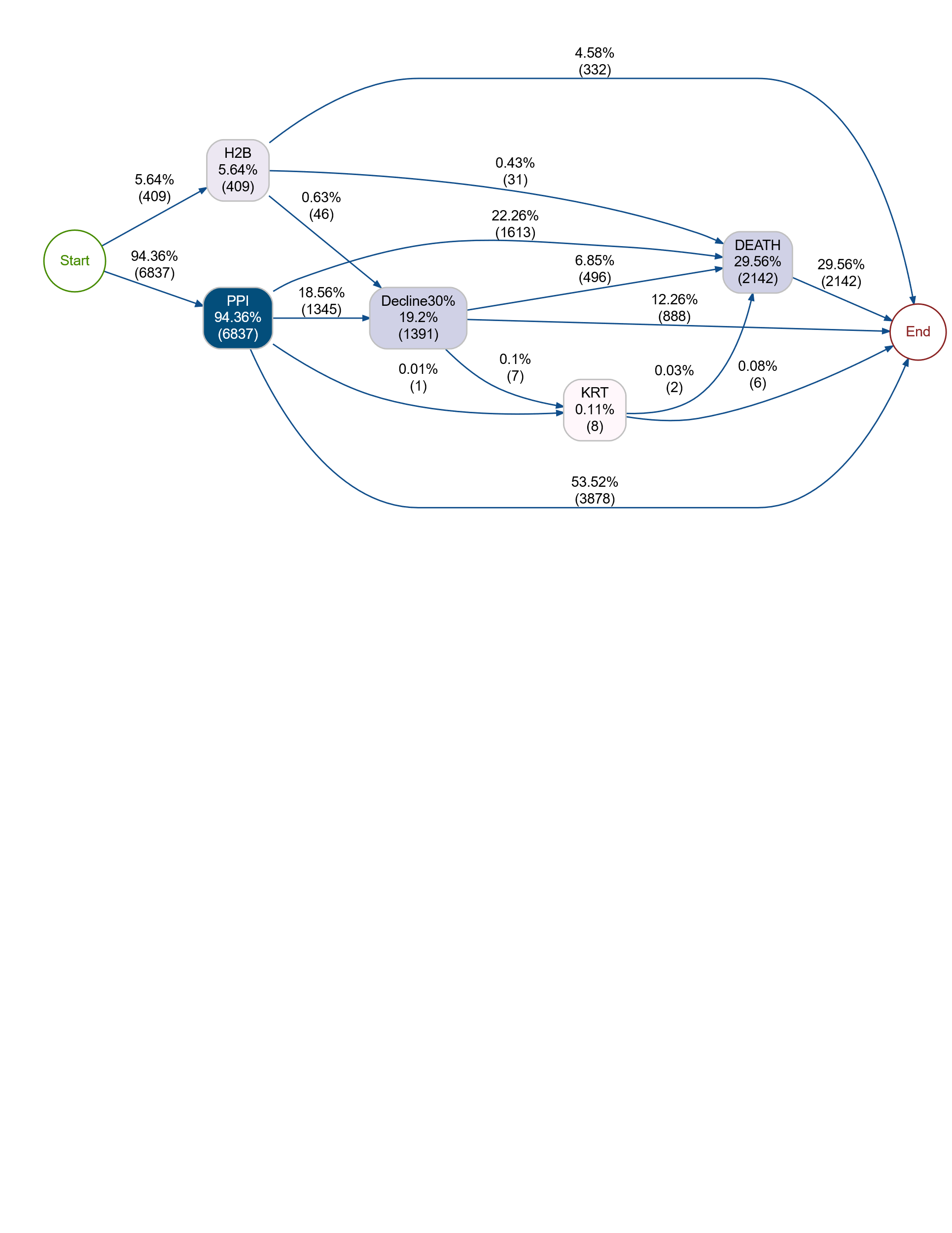
**
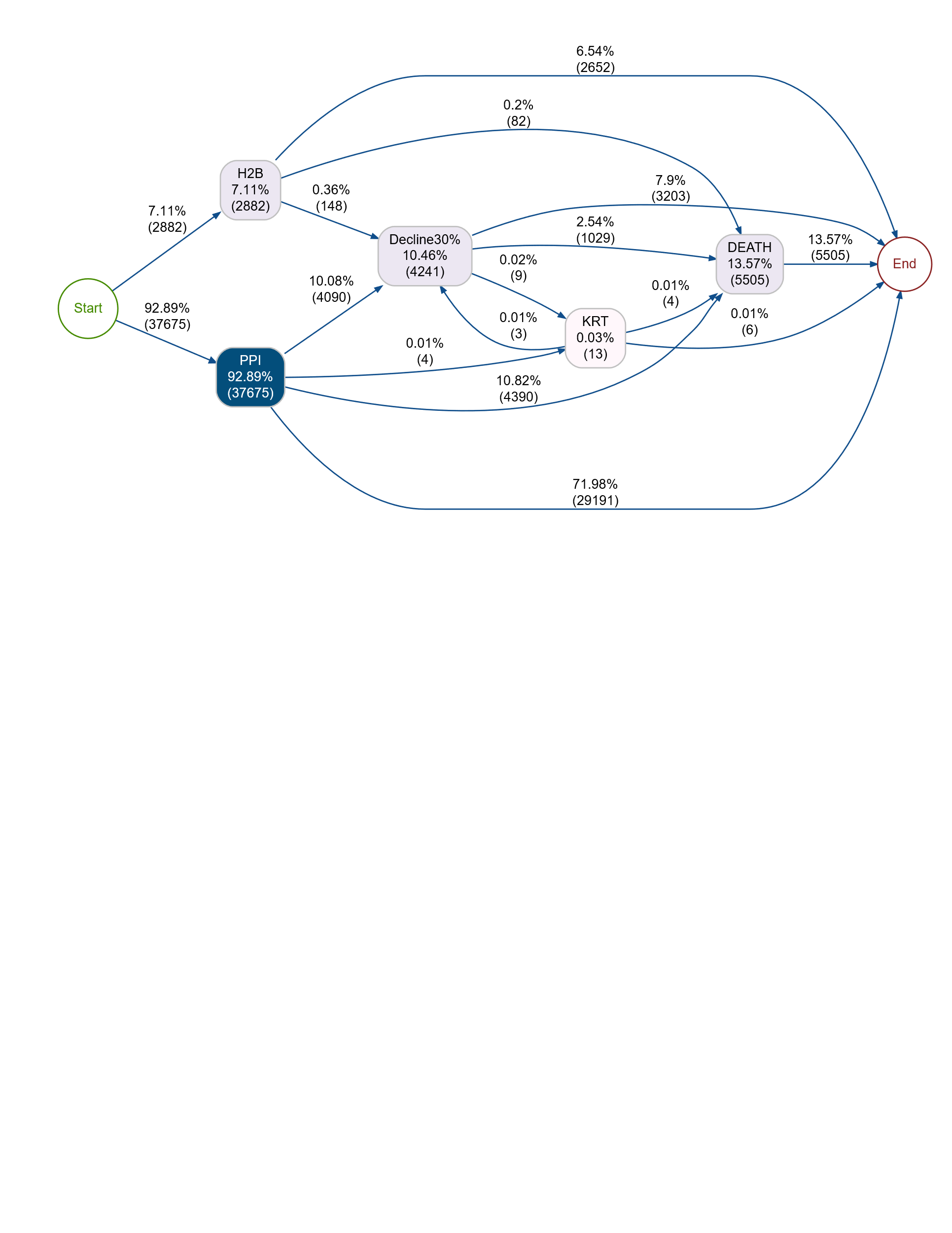
***
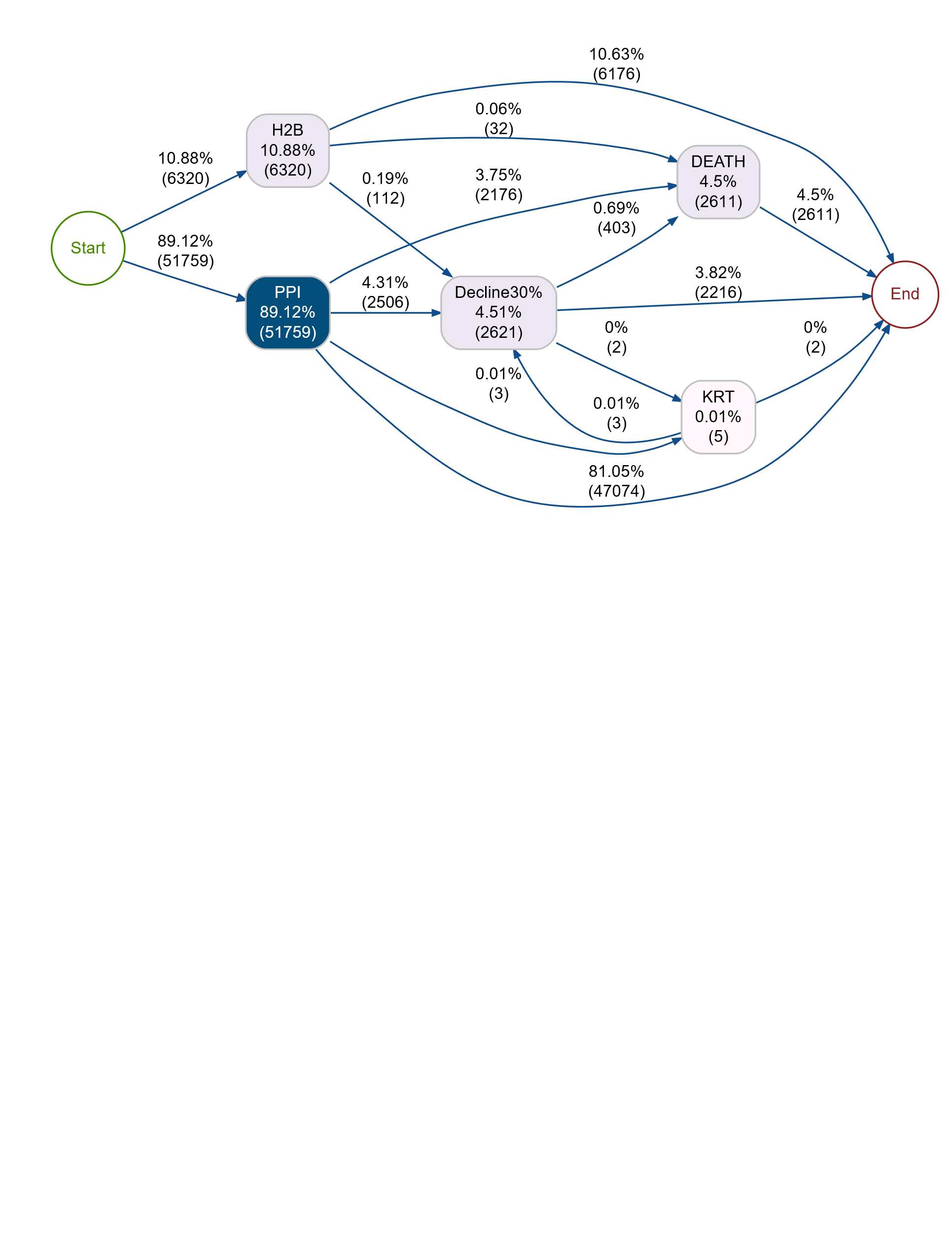
***

eGFR 3A

eGFR 3B

**
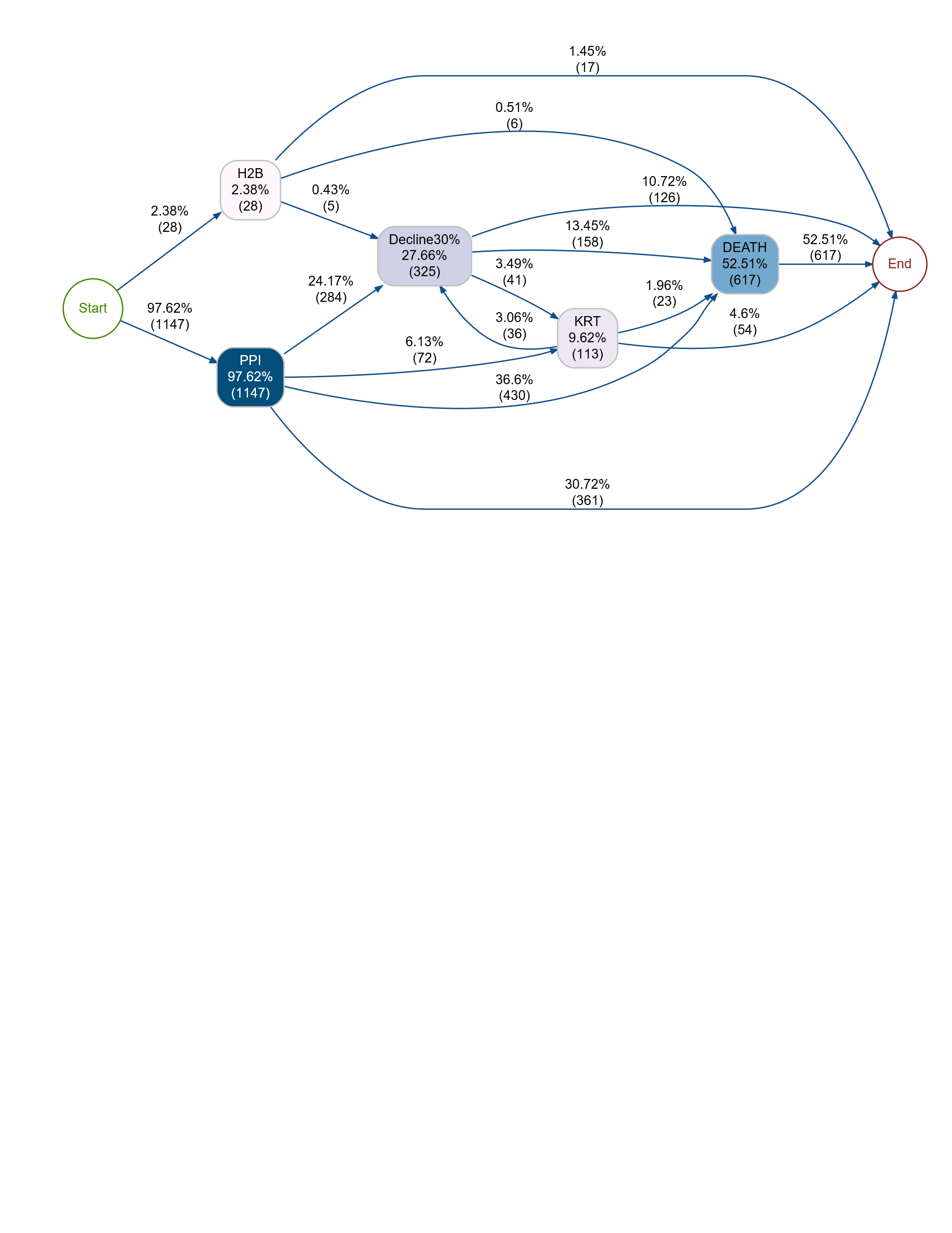
**

eGFR 4
